# Calpains as novel players in the molecular pathogenesis of spinocerebellar ataxia type 17

**DOI:** 10.1007/s00018-022-04274-6

**Published:** 2022-04-28

**Authors:** Jonasz Jeremiasz Weber, Stefanie Cari Anger, Priscila Pereira Sena, Rana Dilara Incebacak Eltemur, Chrisovalantou Huridou, Florian Fath, Caspar Gross, Nicolas Casadei, Olaf Riess, Huu Phuc Nguyen

**Affiliations:** 1 Department of Human Genetics, Ruhr University Bochum, Universitätsstraße 150, 44801 Bochum, Germany; 2 Institute of Medical Genetics and Applied Genomics, University of Tübingen, 72076 Tübingen, Germany; 3 Graduate School of Cellular Neuroscience, University of Tübingen, 72074 Tübingen, Germany; 4 NGS Competence Center Tübingen, 72076 Tübingen, Germany

**Keywords:** TATA box-binding protein, Spinocerebellar ataxia type 17, Polyglutamine diseases, Calpains, Proteolytic cleavage, Transcriptome

## Abstract

Spinocerebellar ataxia type 17 (SCA17) is a neurodegenerative disease caused by a polyglutamine-encoding trinucleotide repeat expansion in the gene of transcription factor TATA box-binding protein (TBP). While its underlying pathomechanism is elusive, polyglutamine-expanded TBP fragments of unknown origin mediate the mutant protein’s toxicity. Calcium-dependent calpain proteases are protagonists in neurodegenerative disorders. Here, we demonstrate that calpains cleave TBP, and emerging C-terminal fragments mislocalize to the cytoplasm. SCA17 cell and rat models exhibited calpain overactivation, leading to excessive fragmentation and depletion of neuronal proteins in vivo. Transcriptome analysis of SCA17 cells revealed synaptogenesis and calcium signaling perturbations, indicating the potential cause of elevated calpain activity. Pharmacological or genetic calpain inhibition reduced TBP cleavage and aggregation, consequently improving cell viability. Our work underlines the general significance of calpains and their activating pathways in neurodegenerative disorders and presents these proteases as novel players in the molecular pathogenesis of SCA17.

**Supplementary Information:**

The online version contains supplementary material available at 10.1007/s00018-022-04274-6.

## Introduction

Spinocerebellar ataxia type 17 (SCA17) is a rare neurodegenerative disorder characterized by ataxia, dementia, involuntary movements such as chorea and dystonia and psychiatric manifestations [1, 2]. Due to its clinical overlaps, SCA17 is also termed Huntington disease (HD)-like 4 (HDL4) [3]. The genetic basis of the disease is a pathological expansion of an imperfect CAG/CAA repeat in the gene encoding the general transcription factor TATA box-binding protein (TBP) [4, 5]. Together with further proteins, the ubiquitously expressed TBP forms the transcription factor IID (TFIID), an element of the RNA polymerase II preinitiation complex [6–8]. CAG/CAA numbers above 42 are considered pathogenic, reaching full penetrance at more than 48 repeats [9–11]. They are translated into an elongated polyglutamine (polyQ) stretch located at the N-terminus of TBP, leading to its aggregation into neuronal intranuclear inclusions [4, 5]. Although the precise molecular mechanism behind polyQ-expanded TBP toxicity is not fully understood, multiple negative ramifications on TBP’s normal function were identified. The polyQ expansion was found to reduce TBP dimerization, compromise the protein’s DNA binding capability, induce aberrant interactions with other polyQ proteins or transcription factors and dysregulate its transcriptional activity [12–14]. Interestingly, a mouse model of SCA17 showed the presence of polyQ-expanded TBP fragments lacking the protein’s C-terminal DNA-binding domain. These cleavage products could readily form nuclear inclusions in cells and cerebellar neurons. Moreover, C-terminally truncated polyQ-expanded TBP is unable to dimerize or bind DNA but sequestered transcription factor IIB (TFIIB) into its aggregates [13, 14]. Interestingly, in Alzheimer disease (AD) brain tissue, an N-terminal TBP fragment was also shown to accumulate, whose presumptive origin was linked to a naturally occurring splice variant [15, 16].

Fragmentation of disease proteins has been observed in multiple neurodegenerative diseases and interpreted not only as a concomitant but also as a relevant mediator of mutant protein toxicity. Several classes of proteolytic enzymes, such as caspases and calpains, are known to cleave disease proteins, releasing harmful and aggregation-prone breakdown products [17, 18]. However, initial investigations on TBP proteolysis using purified caspases or apoptotic cell extracts could not detect the respective fragments [19].

Due to limited knowledge on TBP fragmentation and the lack of a clear association with proteolytic enzymes, we hypothesized that the SCA17 disease protein is a substrate for calpains, a class of calcium-dependent cysteine proteases [20]. These proteases have been linked previously to the generation of toxic fragments of many disease-causing proteins, including the polyQ proteins ataxin-3 in Machado–Joseph disease (MJD) and huntingtin in HD, as well as α-synuclein in Parkinson disease (PD), TDP-43 in amyotrophic lateral sclerosis (ALS) or tau in AD [21–26]. Furthermore, calpains are known to be overactivated in various neurodegenerative conditions, triggering cellular dysfunctions that may eventually lead to neuronal demise [27–33]. Consequently, targeting calpain activity by pharmacological or genetic strategies was demonstrated to be a potential therapeutic approach in animal and cell models of AD, ALS, HD, MJD, spinal muscular atrophy (SMA) and PD [27–29, 33–38].

To test our hypothesis, we triggered calpain-mediated cleavage of wild-type and polyQ-expanded TBP in vitro and in cells and compared the arising fragmentation pattern and its intracellular localization with TBP’s physiological proteolysis. Moreover, we investigated if the calpain system is overactivated in SCA17 cells or in our TBPQ64 rats [39] and evaluated the repercussions on neuronal calpain substrate proteins. By performing transcriptome analysis of SCA17 cells, we looked at alterations of molecular signaling pathways triggered by polyQ-expanded TBP that potentially account for calpain activation and neuronal dysfunction. Finally, we lowered calpain activity by pharmacological means and by overexpressing its proteinaceous inhibitor calpastatin (CAST) in SCA17 cells to assess possible consequences on TBP cleavage and aggregation as well as on cell viability.

## Materials and methods

### Animals and brain tissue sampling

Wild-type and TBPQ64 transgenic rats, previously generated in our institute and expressing full-length mutant human TBP with 64 glutamines predominantly in cerebellum [39], were kept under standard housing conditions and 12 h light/dark cycle until the age of 10 months, which represents the terminal stage of this model’s severe phenotypic disease progression. For protein extraction, animals were killed by CO_2_ inhalation and brains were immediately dissected on ice, snap-frozen in liquid nitrogen and stored at –80 °C for further analysis.

### Expression constructs

For overexpression of human TBP, pBSSKII + mPrP vectors carrying cDNA for myc-tagged full-length TBP with 38 or 64 glutamines [39] and pCMS-EGFP vectors carrying cDNA for TBP with 13 or 105 glutamines were employed [13]. Overexpression of calpastatin was achieved using a pRK5 vector carrying the cDNA for full-length human calpastatin (hCAST) [40].

### Cell culture

For cell culture experiments, HEK 293T cells (ATCC: CRL-11268) were cultured in Dulbecco’s modified Eagle’s medium (DMEM) supplemented with 10% (v/v) fetal bovine serum (FBS), 1% (v/v) non-essential amino acids (MEM NEAA) and 1% (v/v) Antibiotic–Antimycotic (A/A) (all Gibco^®^, Thermo Fisher Scientific) in 5% CO_2_ at 37 °C.

PC12 cells stably expressing TBP featuring 13Q or 105Q [13] were provided by Prof. Shihua Li (Guangdong-Hongkong-Macau Institute of CNS Regeneration, Ministry of Education CNS Regeneration Collaborative Joint Laboratory, Jinan University, 510632, Guangzhou, China) and maintained in DMEM supplemented with 10% (v/v) horse serum, 5% (v/v) FBS, 1% (v/v) G418 and 1% (v/v) A/A (all Gibco^®^, Thermo Fisher Scientific). Transfection of HEK 293T cells was conducted for 48 h or 72 h using the Attractene reagent (Qiagen) according to the manufacturer’s protocol.

### Cell viability assay

For assessing cell viability, HEK 293T cells were seeded in 6-well cell culture plates and transfected 24 h later. 48 h post-transfection, cells were transferred in fresh medium to a 96-well plate (5 wells per condition) and cultured for another 24 h. Cell viability was assessed using the PrestoBlue™ Cell Viability Reagent (Thermo Fisher Scientific), according to the manufacturer’s protocol. Briefly, culture medium was aspirated, and cells were incubated in fresh medium containing the resazurin-based PrestoBlue™ solution in a 1:10 ratio under standard culture conditions for 30 min. Fluorescence signals were measured at 535 nm (excitation)/615 nm (emission) using a Synergy HT plate reader and the Gen5 software (both Biotek).

### Cell-based calpain assays

Cell-based calpain activation assays were performed as previously described [26] using transiently transfected HEK 293T cells or stably transfected PC12 cells. To activate endogenously expressed calpains, cell medium was aspirated and replaced by Opti-MEM^®^ I Reduced Serum Media (Gibco®, Thermo Fisher Scientific) containing 1 µM of the calcium ionophore ionomycin (in DMSO) (407950, Merck Millipore) and 5 mM CaCl_2_ at standard culture conditions for 2 h or indicated time points. For negative controls, cells were pre-incubated with Opti-MEM^®^ I Reduced Serum Media containing 25 µM calpain inhibitor III (CI-III; carbobenzoxy-valinyl-phenylalaninal; in DMSO) (Merck Millipore) 1 h before ionomycin treatments. DMSO served as vehicle control.

For calpain inhibition assays, PC12 cells were solely treated with 25 µM calpain inhibitor CI-III in standard medium for 24 h or 48 h. When treated for 48 h, medium was replenished by fresh medium and compound after 24 h.

### Immunostaining and fluorescence microscopy

For immunocytochemistry staining, HEK 293T cells were seeded on 8-well chamber slides (80841, Ibidi), transfected with respective constructs and cultured for 72 h. Subsequently, cells were pre-fixed by supplementing the medium with 0.4% (w/v) PFA and incubating at 37 °C for 10 min. Afterwards, medium was aspirated, cells were washed once with 1 × DPBS (Gibco^®^, Thermo Fisher Scientific) and incubated in 4% (w/v) PFA in 1 × DPBS for 15 min. Next, fixative was removed, cells were washed three times with 1 × DPBS for 5 min and incubated in blocking/permeabilization buffer (10% (w/v) BSA, 0.5% (v/v) Triton X-100, 0.02% (w/v) NaN_3_ in 1 × DPBS) at room temperature for 1 h. Cells were probed with rabbit anti-calpain-1 (1:500; CSB-PA004490ESR2HU, Cusabio Technology), mouse anti-calpain-2 (1:500; clone 1E1F10, 66977–1-Ig, Proteintech), rabbit anti-calpastatin (1:50; H-300, sc-20779, Santa Cruz Biotechnology), mouse anti-polyglutamine/TBP (1:1000; 5TF1-1C2, MAB1574, Merck Millipore) or rabbit anti-TBP (1:50; N-12, sc-204, Santa Cruz Biotechnology) antibodies in antibody diluent (1% (w/v) BSA, 0.5% (v/v) Triton X-100, 0.02% (w/v) NaN_3_ in 1 × DPBS) at 4 °C overnight. The next day, cells were washed four times with 1 × DPBS and incubated with goat anti-mouse or rabbit Alexa Fluor 488 or 555 (all 1:500; A32723, A27034, A28180, or A32732, all Thermo Fisher Scientific) secondary antibodies at room temperature for 1 h. After washing four times with 1 × DPBS for 5 min, the media chamber was removed, cells mounted with VECTASHIELD^®^ Antifade Mounting Medium with DAPI (H-1200, Vector Laboratories) using coverslips and sealed with transparent nail polish.

Fluorescent images were taken at a 200 × magnification on an Axioplan 2 imaging microscope equipped with an ApoTome, a Plan-Neofluar 20 × /0.50 objective and an AxioCam MRm camera, using the AxioVision 4.3 imaging software (all Zeiss).

### Protein extraction

Tissue homogenates and lysates were obtained by homogenizing rat cerebella in RIPA buffer (50 mM Tris pH 7.5, 150 mM NaCl, 0.1% (w/v) SDS, 0.5% sodium deoxycholate and 1% (v/v) Triton X-100) containing cOmplete™ protease inhibitor cocktail (Roche) using an ULTRA-TURRAX^®^ disperser (VWR). Homogenates were incubated for 25 min on ice and stored at −80 °C for further analysis. For tissue lysate preparation, homogenates were centrifuged for 30 min at 4 °C and 16,100×*g*. The supernatant was transferred into a fresh, pre-cooled reaction tube and mixed with glycerol to a final concentration of 10% (v/v).

Cell homogenates and lysates were prepared as follows: HEK 293T and PC12 cells were dissociated by gentle pipetting and transferred to 1.5 ml tubes. Cell pellets were obtained by centrifugation at 300×*g* for 5 min followed by aspiration of the supernatant. Pellets were washed once with cold 1 × DPBS. Homogenization was conducted by resuspending the cell pellet in RIPA buffer containing cOmplete™ protease inhibitor cocktail and ultrasonication using a Sonopuls ultrasonic homogenizer (Bandelin) for 5 s and 50% pulse duration at 10% power. Homogenates were mixed with glycerol to a final concentration of 10% (v/v). For cell lysate preparation, homogenates were centrifuged for 10 min at 4 °C and 16,100×*g*. Supernatants were mixed with glycerol to a final concentration of 10% in a fresh, pre-cooled reaction tube.

Protein concentrations of homogenates and lysates were measured spectrophotometrically in a microtiter plate using Bradford reagent (Bio-Rad Laboratories). Samples were stored at −80 °C until further analysis.

### Cytoplasmic-nuclear fractionation

For separation of cytoplasmic and nuclear proteins, an *enhanced Rapid, Efficient And Practical* (eREAP) fractionation method was applied based on the original protocol by Suzuki et al*.*, with adjustments described previously [26, 41]. For the enhanced method, pellets of the nuclear fraction were resuspended in DPBS-N (DPBS, 0.1% (v/v) NP-40, containing cOmplete™ protease inhibitor) followed by ultrasonication at a 50% pulse duration and 10% power for 3 s. All fraction volumes were equalized. For reaching an improved comparability between samples, protein concentrations of obtained fractions were measured using the Bradford method and equal protein amounts were loaded for western blot analysis.

### Calpain cleavage assays in vitro

In vitro-calpain cleavage assays were performed with 25 µg protein amount of rat cerebellar tissue or HEK 293T cell homogenates after lysis in calpain reaction buffer (CRB) (20 mM HEPES/KOH pH 7.6, 10 mM KCl, 1.5 mM MgCl_2_, 1 mM dithiothreitol) and determination of the protein concentration using Bradford reagent. Control and time series samples were diluted in CRB and incubated with 2 mM CaCl_2_ and 50–200 ng of purified calpain-1 (208712) or calpain-2 (208718) (both Merck) in a total volume of 20 µl at room temperature. Calpain inhibition was achieved by pre-incubation with 0.5 mM CI-III. Reactions were stopped at time points 0, 5, 10, 15 and 30 min by addition of 4 × LDS buffer (1 M Tris pH 8.5, 50% (v/v) glycerol, 8% (w/v) LDS, 2 mM EDTA, 0.1% (w/v) Orange G) in a ratio 3:1 plus 100 mM dithiothreitol, and heat-denaturing at 70 °C for 10 min. All samples were further analyzed via western blotting.

### Western blotting

Western blotting was performed according to standard procedures. Briefly, 25 or 30 µg of protein from tissue/cell homogenates or lysates was mixed with a 4 × LDS sample buffer in a ratio 3:1 and supplemented with 100 mM dithiothreitol. After heat-denaturing for 10 min at 70 °C, protein samples were electrophoretically separated using homemade 10% Bis–Tris gels and MOPS electrophoresis buffer (50 mM MOPS, 50 mM Tris pH 7.7, 0.1% (w/v) SDS, 1 mM EDTA) or pre-cast 7% NuPAGE Tris–acetate gels (Thermo Fisher Scientific) and Tris–acetate running buffer (50 mM Tricine, 50 mM Tris pH 8.25, 0.1% (w/v) SDS). Proteins were transferred on Amersham™ Protran™ Premium 0.2 µm nitrocellulose or Amersham™ Hybond^®^ LFP PVDF membranes (both Cytiva) using Bicine/Bis–Tris transfer buffer (25 mM Bicine, 25 mM Bis–Tris pH 7.2, 1 mM EDTA, 15% (v/v) methanol) and a TE22 Transfer Tank (Hoefer) at 80 V and a maximum of 250 mA for 1.5 h.

After transfer, membranes were blocked for 1 h with 5% (w/v) skim milk powder (Sigma-Aldrich) in TBS (10 mM Tris pH 7.5, 150 mM NaCl) at room temperature and probed overnight at 4 °C with primary antibodies diluted in TBS-T (TBS with 0.1% (v/v) Tween 20). A detailed listing of applied primary antibodies can be found in Supplementary Table S1, Supplementary File 1. Afterwards, membranes were washed with TBS-T and incubated at room temperature for 1 h with the respective secondary IRDye^®^ antibodies donkey anti-goat 800CW (P/N 926-32214), goat anti-mouse 680LT (P/N 926-68020), goat anti-mouse 800CW (P/N 926-32210) and goat anti-rabbit 800CW (P/N 926-32211) (all 1:10,000; LI-COR Biosciences). After final washing with TBS-T, fluorescence signals were detected using the LI-COR ODYSSEY^®^ FC and quantified with Image Studio 4.0 software (both LI-COR Biosciences).

### Filter retardation assay

Detection of SDS-insoluble TBP was performed based on the previously described assay for polyQ-expanded ataxin-3 [26]. Briefly, for cell culture samples, 1.5 µg (HEK 293T) or 25 µg (PC12) total protein of cell homogenates was diluted in DPBS containing 2% (w/v) SDS and 50 mM dithiothreitol. Afterwards, samples were heat-denatured at 95 °C for 5 min and filtered through a cellulose acetate membrane (0.2 µm, OE66, GE Healthcare) using a Minifold^®^ II Slot Blot System (Schleicher & Schuell). For rat brain samples, 5 µg total protein of tissue homogenates was filtered through an Amersham™ Protran™ nitrocellulose membrane (0.45 µm, GE Healthcare). Afterwards membranes were detected using the primary antibody rabbit anti-TBP (1:500; N-12, sc-204, Santa Cruz Biotechnology) and secondary antibody goat anti-rabbit 800CW (1:5000; LI-COR Biosciences). Fluorescence signals were detected using the LI-COR ODYSSEY^®^ FC and quantified with Image Studio 4.0 software (both LI-COR Biosciences).

### RNA sequencing

RNA isolation from PC12 cells was conducted using the RNeasy mini (Qiagen) following the manufacturer’s instructions. More precisely, cells were lysed in an appropriate volume of RLT buffer using QIAshredder. DNA digestion was performed on column using RNase-Free DNase Set (Qiagen). RNA was eluted in 30 µl of RNase free water.

RNA quality was determined by measuring the 260/280 and 230/260 absorbance ratio on a spectrophotometer (Nanodrop ND-1000, PEQLAB), the RNA concentration using the Qubit Fluorometric Quantitation and RNA Broad-Range Assay (Thermo Fisher Scientific) and RNA Integrity Number RIN using the Fragment Analyzer 5300 and the Fragment Analyzer RNA kit (Agilent Technologies). For library preparation, mRNA fraction was enriched using oligodT priming from 100 ng of total RNA using the QuantSeq 3' mRNA-Seq (Lexogen). Next, mRNA libraries were prepared according to the manufacturer’s instructions using the UMI Second Strand Synthesis Module and library amplification was performed with 15 PCR cycles. Library molarity was determined by measuring the library size (broad peak of approximately 160–400 bp) using the Fragment Analyzer 5300 and the Fragment Analyzer DNA HS NGS fragment kit (Agilent Technologies), and the library concentration (> 1 ng/µl) using Qubit Fluorometric Quantitation and dsDNA High sensitivity assay (Thermo Fisher Scientific). Libraries were denatured according to the manufacturer’s instructions, diluted to a concentration of 800 pM and sequenced as dual 100 bp reads on an Illumina NovaSeq 6000 (Illumina) with a sequencing depth > 10 million clusters per sample. Library preparation and sequencing procedures were performed by the same individual, and a design aimed to minimize technical batch effects was chosen.

Read quality of RNA sequencing data in fastq files was assessed using ngs-bits (v2020_12-60) to identify sequencing cycles with low average quality, adaptor contamination, or repetitive sequences from PCR amplification. Reads were aligned using STAR (v2.7.3a) allowing gapped alignments to account for splicing against a custom-built genome composed of the Ensembl rat Rnor6. Alignment quality was analyzed using samtools (v1.11) and visually inspected in the Integrative Genome Viewer (v2.7.2). Normalized read counts for all genes were obtained using TMM normalized expression in edgeR (v3.32.0) on R (v4.0.2). Transcripts covered with less than 10 counts in at least three samples were excluded from the analysis using the function with default filter “filterByExpr” leaving > 9,000 genes for determining differential expression in each of the pair-wise comparisons between experimental groups.

Distribution of logarithmized cpm-normalized expression values showed similar characteristics over all samples. Based on the filtered data set, samples were investigated with respect to their pairwise similarity. Spearman’s rank correlation coefficient was calculated for each pair of samples. A hierarchical clustering was performed on the resulting similarity values. Differential gene expression analysis was conducted based on the filtered gene expression data set. A statistical model incorporating the group property of samples was tested by fitting a negative binomial distribution using a generalized linear model (GLM) approach. For each gene, gene expression fold changes (log_2_ fold change) were computed, and a statistical test based on quasi-likelihood (QL) method and using a generalized linear model of the function glmQLFit of edgeR was performed to assess the significance, which is given as a raw *p*-value and adjusted *p*-value (FDR, obtained by Benjamini–Hochberg procedure).

### Reverse transcription quantitative real-time PCR

Reverse transcription of 1 µg RNA into cDNA was performed using the QuantiTect Reverse Transcription Kit (Qiagen) according to the manufacturer’s instructions. Differentially expressed genes *Atp2a3*, *Calm2*, *Cdh2*, *Gria2*, Ryr2 and *Syt11*, were validated by quantitative real-time PCR (qPCR) using the SYBR Green PCR Master Mix (Qiagen). In particular, a reaction mixture of 1 × QuantiTect SYBR Green PCR Master Mix, 1 µM forward and 1 µM reverse primer (metabion international AG; Suppl. Table S2, Supplementary File 1) as well as RNase free water was added to cDNA (1:50). qPCR was run on LightCycler 480 II (Roche) using LightCycler 480 Software 1.5.62 SP3 following the manufacturer’s protocol. For normalization, reference genes *Actb*, *Pgk1* and *Ubc* were used.

### Pathway analysis

For canonical pathway enrichment, differentially expressed genes (DEGs) obtained from RNA sequencing were analyzed using the core analysis platform of the Ingenuity Pathway Analysis (IPA) software (v60467501) (Qiagen). Suggested default parameters were used and DEGs with a log_2_ fold change (log_2_fc) value ≥ 1 and *p*-value ≤ 0.01 were taken into consideration. Results were graphically visualized using GraphPad Prism 8.40 (GraphPad Software).

### In silico prediction tools

Computational analyses of features within the TBP sequence were based on the canonical isoform 1 of human TBP (UniProt identifier: P20226-1). For in silico calpain cleavage site prediction the Group-based Prediction System—Calpain Cleavage Detector (GPS-CCD) tool was employed (available at http://ccd.biocuckoo.org/), which is based on 368 experimentally verified calpain cleavage sites in 130 proteins [42]. Scores for every amino acid position were graphically visualized via GraphPad Prism 8.40 (GraphPad Software). For prediction of nuclear export signals (NES), the NetNES tool (available at http://www.cbs.dtu.dk/services/NetNES/) was used, which predicts leucine-rich NES based on a combination of neural networks and hidden Markov models [43].

### Statistical analysis

Data were statistically analyzed using GraphPad Prism 8.40 (GraphPad Software). Results are presented as mean + SEM. One-sample *t*-test, Student’s *t*-test, or one-way ANOVA with Dunnett’s post hoc analysis was applied. Where necessary, *p*-values were corrected for multiple testing via the Hommel method using R (v3.1.3) with the p.adjust package [44]. Significance was assumed with a *p-*value ≤ 0.05. For nonlinear regression analysis, a one-phase decay model with robust regression fitting method and a plateau constraint = 0 was applied. See respective figure legends for further details.

## Results

### TBP fragments are present in SCA17 cells and rat cerebellum

**Fig. 1:**
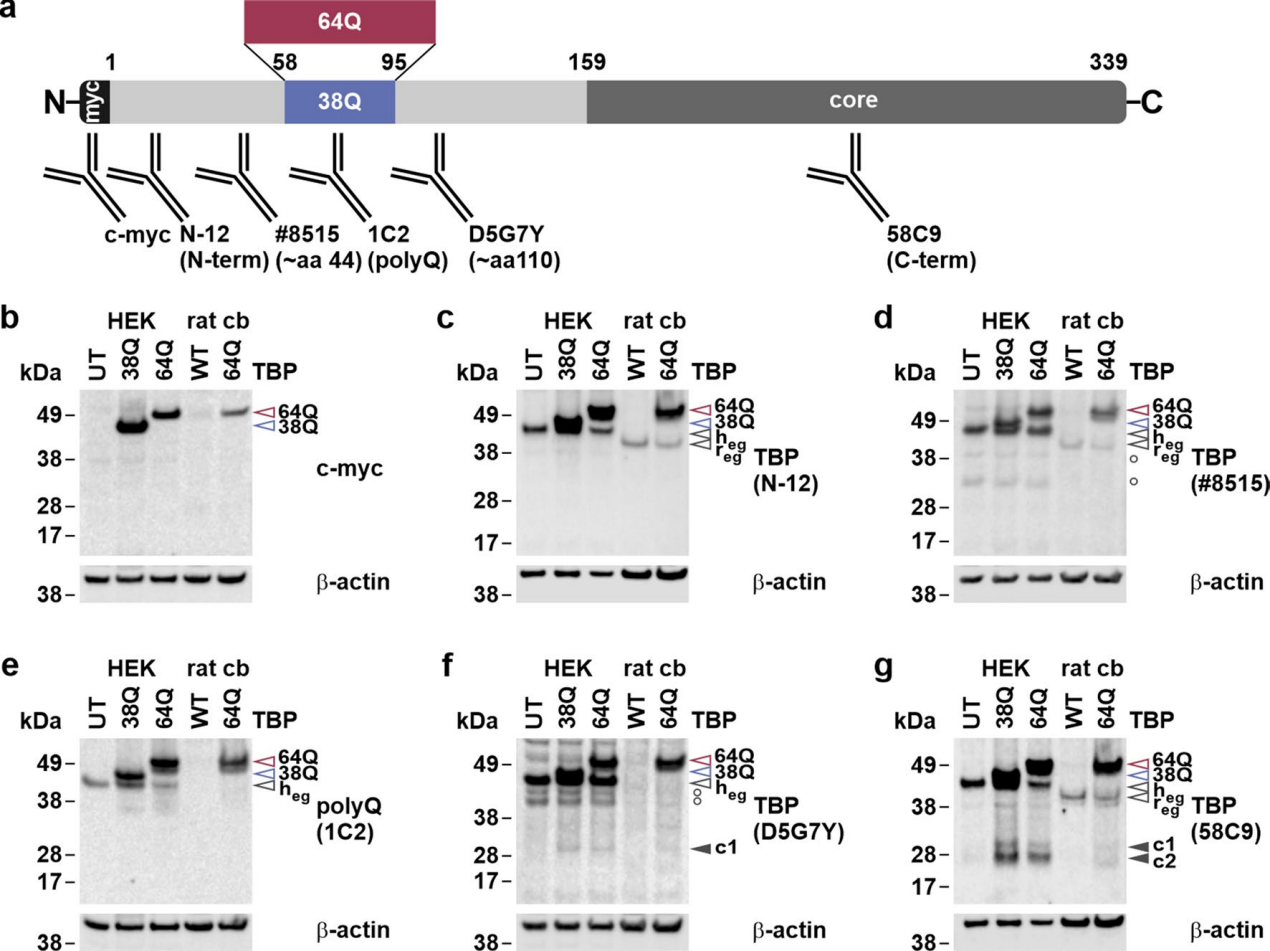
Transfected HEK 293T cells and TBPQ64 rat cerebellum feature C-terminal TBP fragments. **a** Graphic representation of the myc-tagged human full-length TBP (myc-TBP; reference isoform 1; UniProt identifier: P20226-1) with 38Q or 64Q and approximate epitopes of the utilized antibodies. **b–g** Protein extracts of untransfected (UT) and myc-TBP 38Q or 64Q-transfected HEK 293T (HEK) cells, and of wild-type (WT) or TBPQ64 (64Q) rat cerebellum (cb) were assayed by western blotting and immunodetection using a c-myc specific antibody (**b**), the polyQ-specific antibody 1C2 (**e**) and the TBP-specific antibodies N-12 (**c**), #8515 (**d**), D5G7Y (**f**) and 58C9 (**g**). β-actin served as loading control. Except for **c** and **g**, each antibody detection was performed on an individual membrane. Empty arrowheads indicate full-length TBP: red-rimmed = 64Q, blue-rimmed = 38Q, black-rimmed = endogenous human/rat TBP (h_eg_/r_eg_). Black arrowheads indicate C-terminal TBP fragments (c1/c2). White circles (○) indicate unspecific bands

The occurrence of TBP fragments has been reported not only for brain tissue of SCA17 mice, but also of AD patients [14, 15]. To examine whether these fragments are also detectable in our SCA17 models, we performed western blot analysis of protein homogenates extracted from HEK 293T cells transfected with myc-tagged full-length human TBP (myc-TBP) with 38Q or 64Q and from the cerebellum of our TBPQ64 rats. Along with these samples, extracts of untransfected cells and wild-type (WT) rats were assayed as controls. To achieve good coverage of the entire TBP, we applied a selection of antibodies with epitopes distributed from the myc-tagged N-terminus to the C-terminal DNA-binding core domain of the overexpressed protein (Fig. 1a). Immunodetection using the c-myc-specific antibody stained overexpressed full-length TBP in cells and rat cerebellum (Fig. 1b), whereas antibodies N-12, #8515 and 58C9 additionally detected the endogenous full-length protein (Fig. 1c, d, g). Antibody D5G7Y stained all human TBP full-length species in our samples but failed binding to endogenous TBP in rat cerebellum (Fig. 1f). The same applied to polyQ-specific antibody 1C2 (Fig. 1e), which detects polyQ stretches greater than or equal to 38Q.

Two potential C-terminal TBP fragments, c1 (migrating above 28 kDa) and c2 (migrating below 28 kDa), were detected by the antibody 58C9 in both transfected HEK 293T cells and TBPQ64 rat cerebellum (Fig. 1g). These fragments lack the polyQ stretch as they neither presented corresponding size shifts nor were detected by the polyQ-specific antibody 1C2 (Fig. 1e). Untransfected HEK 293T cells and WT rats did not feature both fragment bands, which might be due to the comparably lower expression levels of endogenous TBP. Fragment c1 was also detected by antibody D5G7Y (Fig. 1f), suggesting that it spans from the D5G7Y epitope, located around Ala110 (human reference isoform 1; UniProt identifier: P20226-1), to the C-terminus of TBP, which is detected by antibody 58C9. Surprisingly, none of the other antibodies with specificities for the N-terminal portion of TBP were able to stain further TBP fragments, neither on nitrocellulose nor PVDF membranes (Fig. 1b–e, Suppl. Fig. S1a and b, Supplementary File 1), prompting the question of the fate of the N-terminal, polyQ stretch-containing counterparts of fragments c1 and c2.

### In silico tool predicts multiple calpain cleavage sites within TBP

Previous attempts have failed to associate proteases with the fragmentation of TBP, while an N-terminal fragment was shown to originate from alternative splicing in AD patient brains [16, 19]. As calpains have been associated with the fragmentation of multiple disease proteins in neurodegenerative disorders [21, 23–25, 45], we hypothesized that these enzymes are also accountable for the observed fragments in our models and, consequently, for the cleavage of polyQ-expanded TBP in SCA17.

**Fig. 2:**
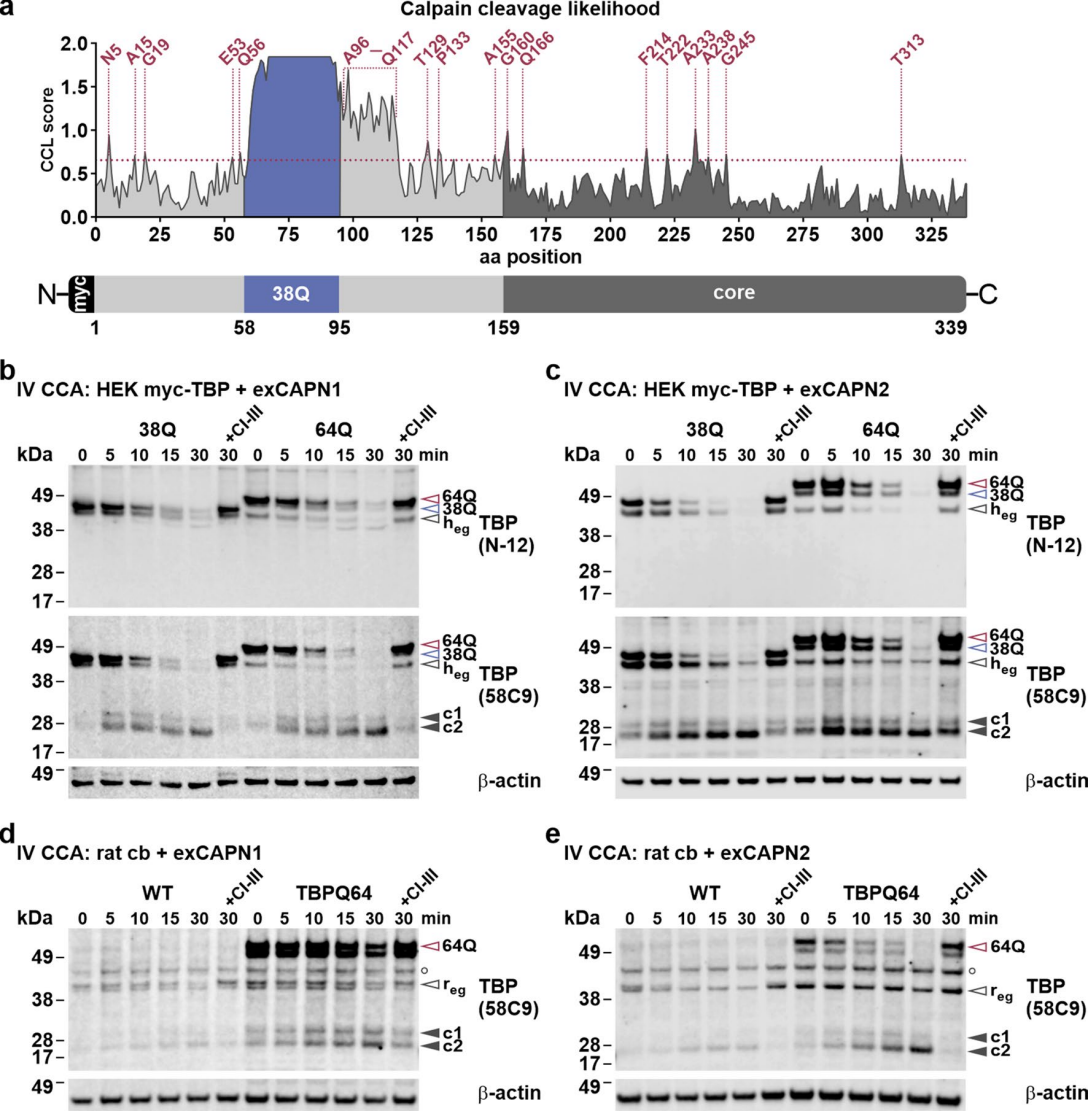
TBP emerges as a calpain substrate from in silico predictions and in vitro cleavage assays. **a** In silico prediction of calpain cleavage sites in the human reference isoform 1 of TBP using the GPS-CCD tool. The graph shows the distribution of calpain cleavage likelihood (CCL) scores along the TBP sequence. The red dotted line represents the maximum default cut-off value of 0.654. High CCL scores of the polyQ stretch (blue) are considered as prediction artifacts. A schematic of TBP with its main features is depicted underneath the graph, and corresponding regions are highlighted in respective colors. **b–e** For in vitro calpain cleavage assays (IV CCA), protein extracts of myc-TBP 38Q or 64Q-transfected HEK 293T (HEK) cells and of wild-type (WT) or TBPQ64 rat cerebellum (cb) were incubated with purified calpain-1 (exCAPN1) or calpain-2 (exCAPN2) for up to 30 min. Calpain inhibitor III (CI-III) controls were performed to validate specificity of the reaction. Samples were analyzed by western blotting and immunodetection using the TBP-specific antibodies N-12 and 58C9. β-actin served as loading control. Empty arrowheads indicate full-length TBP: red-rimmed = 64Q, blue-rimmed = 38Q, black-rimmed = endogenous human/rat TBP (h_eg_/r_eg_). Black arrowheads indicate C-terminal TBP fragments (c1/c2). White circle (○) indicates an unspecific band

Before addressing this question on a biological level, we tested the potential cleavage likelihood of TBP using the in silico calpain cleavage site prediction tool GPS-CCD [42]. Based on the canonical sequence of human TBP (UniProt identifier: P20226-1), the prediction tool localized 20 putative calpain cleavage sites along the entire sequence and a cleavage site cluster in the alanine/glutamine-rich stretch between Ala96 and Glu117, which surpasses the maximum default cut-off value of 0.654 (Fig. 2a). A comprehensive listing of predicted calpain cleavage sites can be found in Supplementary Table S3, Supplementary File 1. Analogously to our previous study on the cleavage of the MJD protein ataxin-3 [26], the polyQ stretch of TBP yielded high cleavage likelihood scores, which may be considered a prediction artefact.

### In vitro cleavage assays indicate TBP as a calpain substrate

Based on our computational approach, TBP cleavage by calpains in a biological system seemed probable. To test this, we performed in vitro calpain cleavage assays by incubating protein extracts of HEK 293T cells expressing myc-TBP 38Q or 64Q and of WT and TBPQ64 rat cerebellum with purified calpain-1 or calpain-2 plus CaCl_2_, for up to 30 min. For reasons of comparability, the reaction speed was adjusted by selecting corresponding amounts of both proteases, based on the cleavage of the calpain substrate α-tubulin [46] (Suppl. Fig. S2a and b, Supplementary File 1). To ascertain the specificity of the reaction, we additionally supplemented 30 min-control samples with calpain inhibitor III (CI-III) to block calpain-dependent cleavage. Western blotting was performed to analyze TBP fragmentation in obtained samples using the previously established antibodies N-12, directed against the N-terminus, and 58C9, binding the C-terminal core domain of TBP. Immunostaining with antibody 58C9 detected the previously observed fragments c1 and c2 in protein extracts of HEK 293T cells expressing myc-TBP 38Q or 64Q. Strikingly, incubation with calpain-1 (Fig. 2b) and calpain-2 (Fig. 2c) showed a time-dependent accumulation of both fragment bands, which was abolished by the co-administration of CI-III. Concurrently, full-length endogenous and overexpressed TBP reduced over time, except for the inhibitor-treated samples. Replication of the experiment using protein extracts of WT and TBPQ64 rat cerebellum incubated with calpain-1 (Fig. 2d) and calpain-2 (Fig. 2e) yielded corresponding results. No differences were found between fragmentation patterns of calpain-1 or calpain-2, pointing to conserved cleavage sites of both protease isoforms within TBP. Moreover, fragments c1 and c2 in both human and rat samples showed comparable sizes, which can be attributed to the nearly perfect interspecies sequence identity of TBP’s C-terminal portion adjacent to the polyQ stretch. Interestingly, after an initial increase until 15 min, fragment c1 appeared to reduce overtime while fragment c2 levels continued to rise. This might indicate a further degradation of c1 into smaller breakdown products like c2, which was substantiated by additional double labelling of c1 by antibodies D5G7Y and 58C9, showing that c2 lacks the D5G7Y’s epitope around Ala110 (Suppl. Fig. S2c, Supplementary File 1). As demonstrated in the previous detections, antibody N-12 stained full-length TBP and verified its time-dependent reduction but failed to prove the occurrence of corresponding N-terminal, polyQ stretch-containing TBP fragments (Fig. 2b, c).

### Cell-based calpain activation leads to fragmentation of wild-type and polyQ-expanded TBP

To confirm our findings on calpain-mediated TBP cleavage in the more physiological context of an intact cell, we performed cell-based calpain assays by incubating HEK 293T cells expressing myc-TBP 38Q or 64Q with the calcium ionophore ionomycin. Treatment with this compound leads to an elevation of cytoplasmic calcium concentrations, thereby activating endogenously expressed calpains [26].

**Fig. 3:**
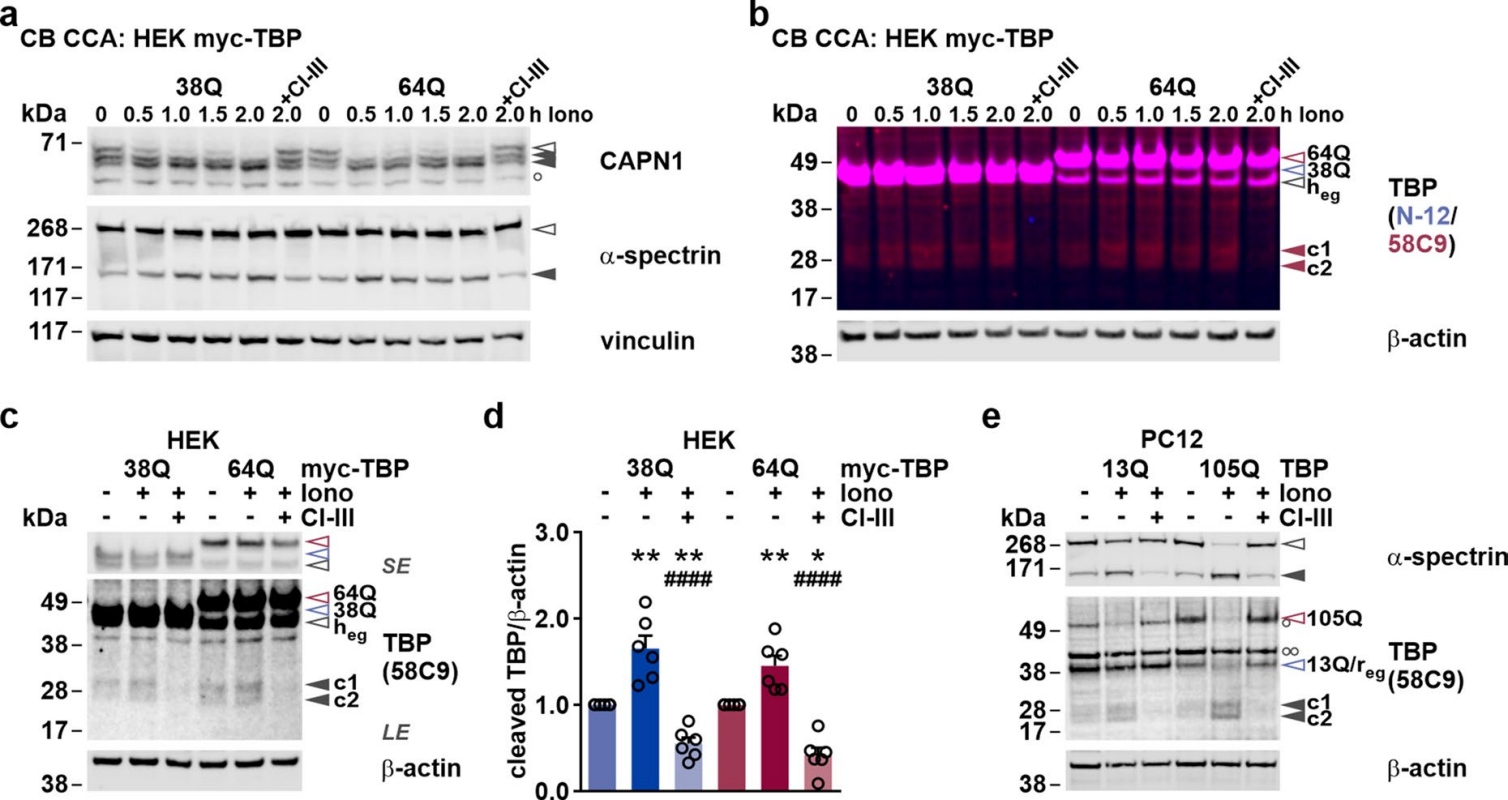
Cell-based calpain activation induces TBP cleavage in cell models of SCA17. **a.** For cell-based calpain cleavage assays (CB CCA), myc-TBP 38Q or 64Q-transfected HEK 293T (HEK) cells were treated with ionomycin (Iono) and CaCl_2_ for up to 2 h. For specificity control, cells were pre-incubated with CI-III for 1 h followed by a co-treatment. Samples were analyzed by western blotting and immunodetection using calpain-1 (CAPN1) and α-spectrin antibodies to assess the degree of ionomycin-induced calpain activation. Vinculin served as loading control. Empty arrowheads indicate full-length proteins. Black arrowheads show respective breakdown products. **b, c** Western blot analysis of ionomycin (Iono)-induced TBP fragmentation was performed using the TBP antibodies N-12 and/or 58C9. Equal loading was confirmed using β-actin. Empty arrowheads indicate full-length TBP: red-rimmed = 64Q, blue-rimmed = 38Q, black-rimmed = endogenous human TBP (h_eg_). Red or black arrowheads indicate C-terminal TBP fragments (c1/c2). *SE* short exposure; *LE* long exposure. **d** Densitometric analysis shows the accumulation of TBP fragments upon ionomycin administration for 2 h or their reduction upon pre-/co-treatment with CI-III. Fragment levels were first normalized to loading control β-actin and then to the respective vehicle-treated control expressing myc-TBP 38Q or 64Q within each assay. Bars represent means + SEM. *n* = 6 repeated assays. *compared to control; ^#^compared to Iono. *p ≤ 0.05, **p ≤ 0.01, ^####^*p* ≤ 0.0001 (one-sample *t*-test or Student’s *t*-test with *p-*value correction for multiple testing via the Hommel method). **e** Cell-based calpain cleavage assays were repeated in PC12 cells expressing TBP with 13Q or 105Q. Samples were analyzed by western blotting and immunodetection using α-spectrin and TBP 58C9 antibodies. Equal loading was confirmed using β-actin. Empty arrowheads indicate full-length proteins: black-rimmed = α-spectrin, red-rimmed = TBP 105Q, blue-rimmed = TBP 13Q and endogenous rat TBP (r_eg_). Black arrowheads indicate the α-spectrin fragment or C-terminal TBP fragments (c1/c2). Single white circle (○) indicates an unspecific band. Two white circles (○○) mark the redetection of loading control β-actin

Transfected HEK 293T cells were incubated with ionomycin plus CaCl_2_ for up to 2 h. For specificity controls, cells were pre-treated with CI-III 1 h before administration of ionomycin. Western blot analysis was performed to investigate the levels of calpain activation and TBP cleavage. First, the effectiveness of the ionomycin-dependent calpain activation was assessed by detecting autoproteolysis of calpain-1 and cleavage of the calpain substrate α-spectrin. Both markers showed increased and time-dependent processing, which was abolished by the pre-incubation with CI-III (Fig. 3a). Immunostaining of TBP using the antibody 58C9 showed a corresponding accumulation of the previously observed fragments c1 and c2 (Fig. 3b). Compared to in vitro calpain cleavage assays, a reduction of full-length TBP was not apparent, as indicated by staining with antibody N-12 and 58C9, which—together with the relatively lower fragment levels—is explained by the lesser degree of calpain-mediated cleavage in living cells. To measure both the levels of induced TBP fragmentation and the efficacy of calpain inhibition by CI-III, we repeated the experiments using a single time point of 2 h (Fig. 3c). Densitometric analysis of the TBP fragment bands c1 and c2 showed a significant 50–60% level increase upon ionomycin treatment, whereas combined pre-incubation and co-treatment with CI-III effectively reduced fragmentation by approximately 50% compared to baseline levels (Fig. 3d). As we sought to reproduce this experiment in a different cell model, we repeated ionomycin and CI-III treatments using a PC12 cell model of SCA17, expressing TBP with 13 or 105Q [13]. Western blot analysis showed similar results for both α-spectrin and TBP fragmentation, including the occurrence of fragment bands c1 and c2, but with observable lowering effects on the respective full-length proteins (Fig. 3e).

Together with the in silico and in vitro analyses, our cell-based assays confirmed TBP as a calpain substrate and calpain-mediated cleavage as a source for the TBP fragments observed at baseline in SCA17 cells and animals.

### C-terminal fragments of TBP accumulate in the cytoplasm

**Fig. 4:**
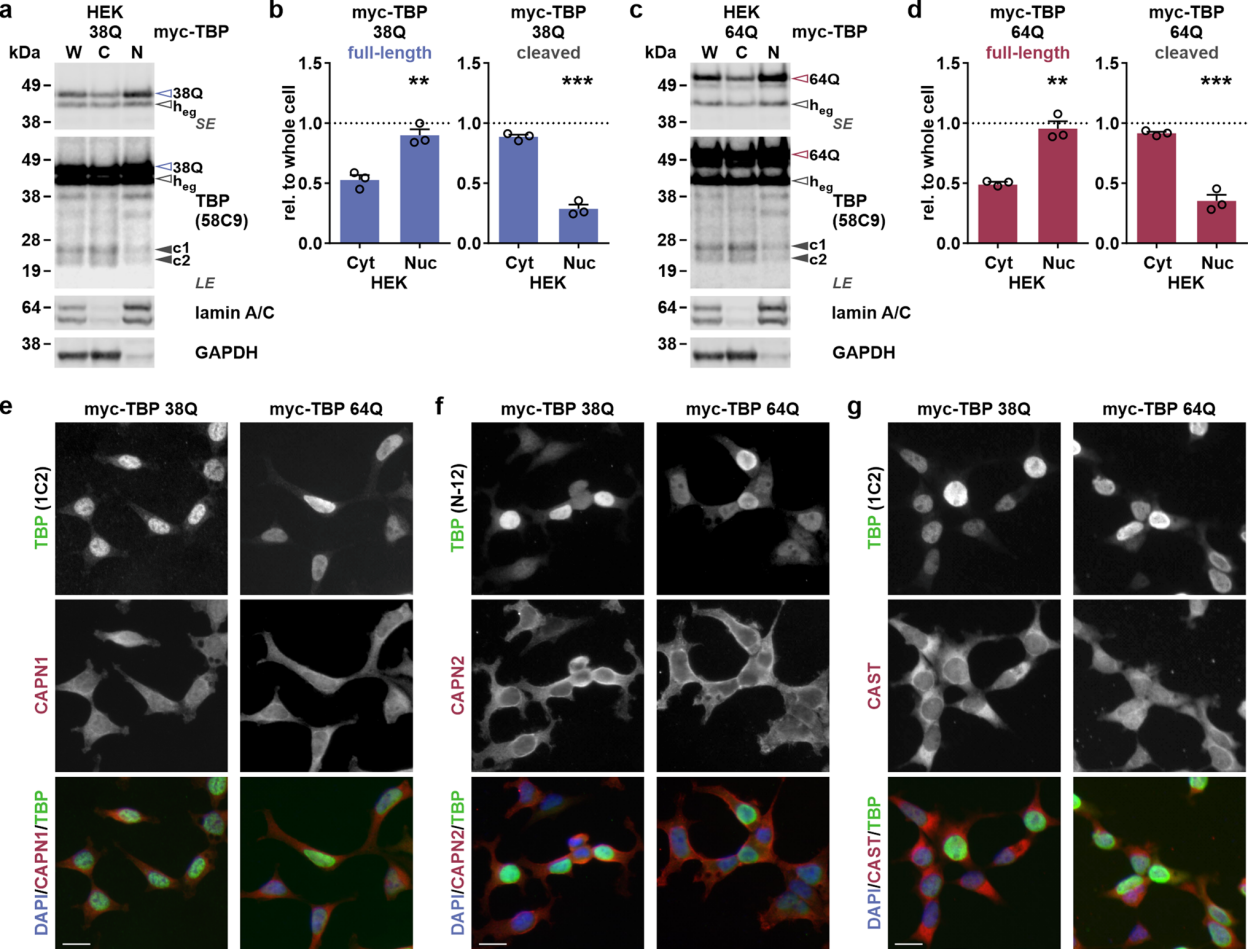
C-terminal TBP fragments are mainly cytoplasmic, sharing localization with members of the calpain system. **a, c** HEK 293T (HEK) cells expressing myc-TBP 38Q or 64Q were subjected to cytoplasmic-nuclear fractionation followed by western blotting. Full-length TBP and its C-terminal fragments c1 and c2 were detected using antibody 58C9. The nuclear proteins lamin A and C, and cytoplasmic protein GAPDH served as fraction purity and loading controls. *W*  whole cell, *C*  cytoplasmic fraction, *N*  nuclear fraction, *LE*  long exposure, *SE*  short exposure. Empty arrowheads indicate full-length proteins: red-rimmed = TBP 64Q, blue-rimmed = TBP 38Q, black-rimmed = endogenous human TBP (h_eg_). Black arrowheads indicate the C-terminal TBP fragments (c1/c2). **b, d** Densitometric analysis of the blots shows levels of full-length myc-TBP and fragments c1 and c2 in the cytoplasmic and nuclear fraction. Levels were normalized to fraction-specific loading control and then relative to (rel. to) whole cell (W) indicated by the dotted line. Bars represent means + SEM. *n* = 3 repeated transfections at different time points. **p ≤ 0.01, ***p ≤ 0.001 (Student’s *t*-test). **e–g** For fluorescence microscopy-based colocalization analysis, HEK 293T (HEK) cells expressing myc-TBP 38Q or 64Q were immunostained using TBP antibodies 1C2 or N-12 together with antibodies against calpain-1 (CAPN1), calpain-2 (CAPN2), or CAST. Appropriate Alexa Fluor 488 (for TBP, green signals)- or Alexa Fluor 555 (for calpains or CAST, red signals)-labeled secondary antibodies were utilized. Nuclei were counterstained with DAPI (blue signals). Acquisitions were made at a 200 × magnification. Scale bars = 20 µm

Previous studies have identified N-terminal, polyQ stretch-containing fragments of TBP in tissues of mice and humans [14, 15]. Interestingly, we could not identify these specific fragments using our approaches but instead robustly detected their potential C-terminal, calpain cleavage-derived counterparts c1 and c2. To scrutinize these fragments’ origin and fate, we sought to determine their intracellular localization, compared to full-length TBP and members of the calpain system. For this, we performed cytoplasmic-nuclear fractionation of untransfected and myc-TBP 38Q or 64Q-transfected HEK 293T cells and screened the obtained fractions for the occurrence of full-length and cleaved TBP via western blotting using antibody 58C9. As expected, we found endogenous and overexpressed TBP having a predominantly nuclear localization (Suppl. Fig. S3a, Supplementary File 1; Fig. 4a–d). A smaller but distinct proportion of the full-length protein was present in the cytoplasmic fraction. In addition, we confirmed the cellular distribution of TBP using microscopy and immunostaining with antibody N-12 (Fig. 4e). Most surprisingly, fragments c1 and c2 showed for both endogenous (Suppl. Fig. S3a, Supplementary File 1) and overexpressed TBP variants with 38Q (Fig. 4a, b) or 64Q (Fig. 4c, d) a localization pattern opposite to the ones of the full-length proteins. To compare localization of TBP with the distribution of members of the calpain system, we likewise performed microscopical (Fig. 4f) and cytoplasmic-nuclear fractionation (Suppl. Fig. S3b, Supplementary File 1) analysis for calpain-1, calpain-2 and CAST. The proteases and their inhibitor exhibited a mainly cytoplasmic localization, although calpain-1 showed an additional characteristic granular occurrence within the nuclei of HEK 293T cells. This suggests that the calpain-mediated fragmentation may rather take place in the cytoplasm. A further hint for active cytoplasmic retention of fragments c1 and c2 was given by performing an in silico prediction for leucine-rich nuclear export signal (NES) using the NetNES tool [43], which indicates a potential NES around amino acid Leu275 within the C-terminus of human TBP (UniProt identifier: P20226-1) (Suppl. Fig. S3c and Suppl. Table S4, Supplementary File 1).

Our fractionation analyses intriguingly demonstrated that the calpain cleavage-derived fragments c1 and c2 are enriched in the cytoplasmic fraction of the cell, which stands in contrast to its source, full-length TBP, but matches the distribution of members of the calpain system, such as calpain-1 and CAST.

### The calpain system is overactivated in SCA17 cells and rat cerebellum

**Fig. 5:**
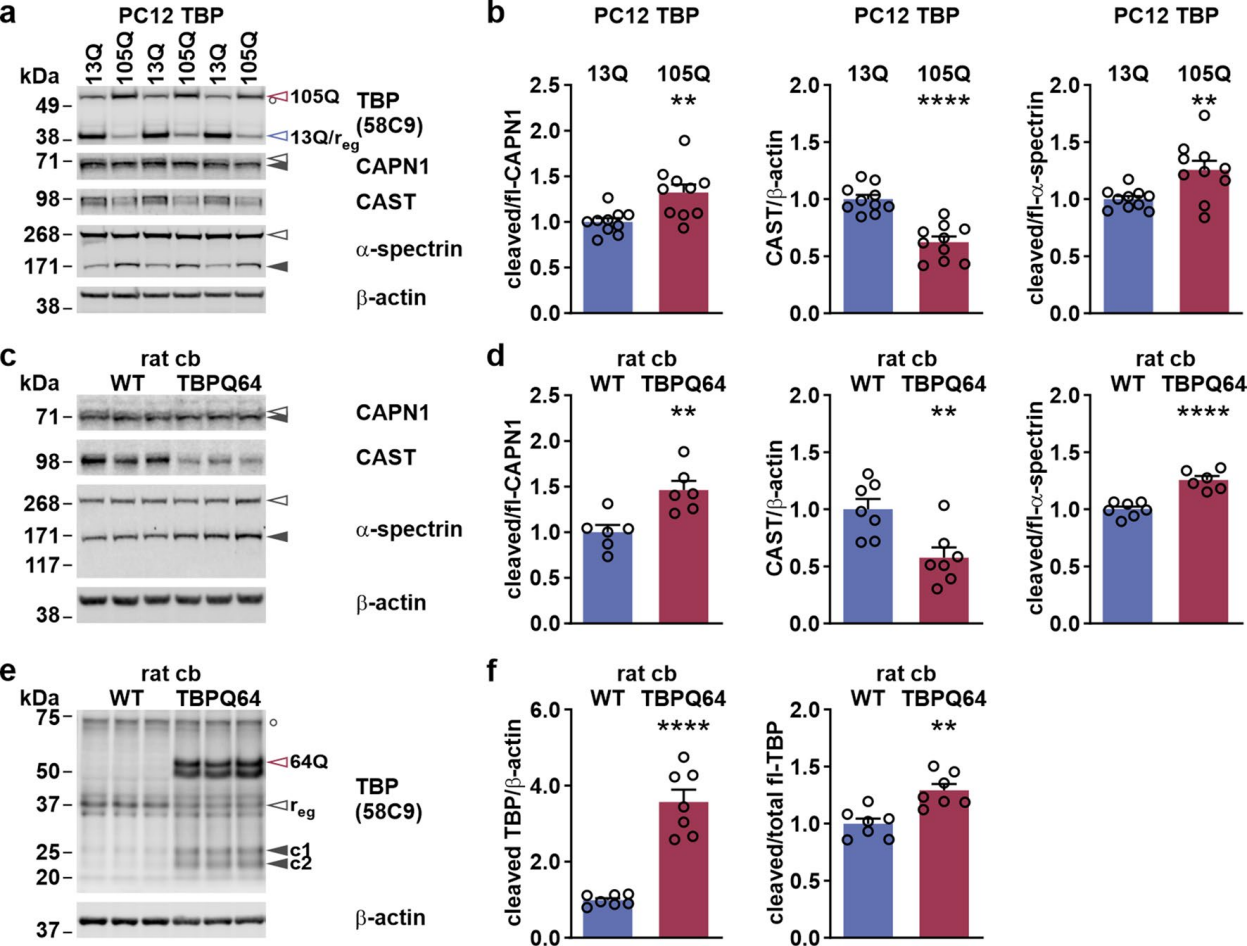
PC12 cell model of SCA17 and TBPQ64 rat cerebellum exhibit calpain overactivation. **a** PC12 cells expressing TBP with 13Q or 105Q were analyzed using western blotting. For assessment of calpain activation, samples were immunodetected with calpain-1 (CAPN1), CAST and α-spectrin antibodies. TBP expression was verified using the TBP 58C9 antibody. Equal loading was confirmed using β-actin. Empty arrowheads indicate full-length proteins: black-rimmed = CAPN1 or α-spectrin, red-rimmed = TBP 105Q, blue-rimmed = TBP 13Q and endogenous rat TBP (r_eg_). Black arrowheads show respective breakdown products. White circle (○) indicates an unspecific band. **b** Densitometric analysis shows CAPN1 cleavage ratio, CAST levels and α-spectrin cleavage ratio. Cleaved CAPN1 and α-spectrin levels were normalized to the respective full-length protein levels, and CAST levels to loading control β-actin. Obtained values were then normalized to the mean values of TBP 13Q-expressing PC12 cells. Bars represent means + SEM. *n* = 10 cell passages per genotype. **p ≤ 0.01, ****p ≤ 0.0001 (Student’s *t*-test). **c** Calpain activation in cerebellum (cb) of WT and TBPQ64 rats was investigated using CAPN1, CAST and α-spectrin antibodies. Equal loading was confirmed using β-actin. Empty arrowheads indicate full-length proteins. Black arrowheads show respective breakdown products. **d** Densitometric analysis shows CAPN1 cleavage ratio, CAST levels and the α-spectrin cleavage ratio. Cleaved CAPN1 and α-spectrin levels were normalized to the respective full-length protein levels, and CAST levels to loading control β-actin. Obtained values were then normalized to the mean values of WT cerebellum. Bars represent means + SEM. *n* = 6–7 animals per genotype. **p ≤ 0.01, ****p ≤ 0.0001 (Student’s *t*-test). **e** TBP fragmentation in TBPQ64 rat cerebellum (cb) was analyzed using the TBP 58C9 antibody. Equal loading was confirmed using β-actin. Empty arrowheads indicate full-length TBP: red-rimmed = 64Q, black-rimmed = endogenous rat TBP (r_eg_). Black arrowheads indicate C-terminal TBP fragments (c1/c2). White circle (○) indicates an unspecific band. **f** Densitometric analysis shows TBP fragment levels and cleavage ratio. Cleaved TBP levels were normalized to loading control β-actin or the full-length TBP. Obtained values were then normalized to the mean values of WT cerebellum. Bars represent means + SEM. *n* = 7 animals per genotype. **p ≤ 0.01, ****p ≤ 0.0001 (Student’s *t*-test)

In parallel to calpain-mediated fragmentation of disease proteins, many neurodegenerative disorders exhibit a pathological overactivation of calpains, which trigger detrimental cascades in affected tissues [47]. To evaluate if this kind of overactivation is present in our SCA17 cell and animal models, we performed western blot analysis of cells expressing TBP with 13Q or 105Q and WT or TBPQ64 rat cerebellum. To assess the state of calpain activation, we detected the autoproteolysis of calpain-1, levels of the endogenous inhibitor CAST and cleavage of the calpain substrate α-spectrin. In PC12 and HEK 293T cells expressing TBP with 105Q, we found significantly reduced CAST levels as well as elevated calpain-1 and α-spectrin fragmentation, pointing towards calpain overactivation (Fig. 5a, b; Suppl. Fig. S4, Supplementary File 1). Next, we analyzed brain tissue of TBPQ64 rats at 10 months of age, which represents a terminal stage of phenotypic progression in our SCA17 animal model [39]. Here, we observed the same significant effects on all three calpain activation markers in the TBPQ64 rat cerebellum compared to WT samples (Fig. 5c, d). Importantly, striatum and cortex of TBPQ64 rats, which feature only very low or low myc-TBP 64Q transgene expression (Suppl. Fig. S5a, Supplementary File 1) [39] and, consequently, lack the SDS-insoluble TBP aggregates as a disease hallmark (Suppl. Fig. S6, Supplementary File 1), showed no evidence of calpain overactivation (Suppl. Fig. S7, Supplementary File 1). These results prove that the expression of a polyQ-expanded TBP drives the elevated activity of calpains. Interestingly, endogenous TBP was reduced upon transgene expression in the TBPQ64 rat cerebellum, pointing to a potential compensatory effect on the regulation of total TBP levels (Suppl. Fig. S5b and c, Supplementary File 1). Moreover, we observed an increased occurrence of fragments c1 and c2 in the TBPQ64 rat cerebellum (Fig. 5e). Fragment levels were approximately threefold higher than in WT tissue, which is mainly due to the transgene-dependent rise in total full-length TBP levels (Suppl. Fig. S5c, Supplementary File 1). However, when normalizing fragment to full-length TBP levels, a significant increase in c1 and c2 levels by approximately 20% was still detectable (Fig. 5f), which might be a consequence of the upregulated calpain activity in TBPQ64 rat cerebellum.

Our findings in PC12 and TBPQ64 rats substantiate calpain overactivation as a new hallmark in models of SCA17, potentially causing further consequences on its molecular pathology.

### Calpain overactivation leads to cleavage and dysregulation of synaptic proteins

**Fig. 6:**
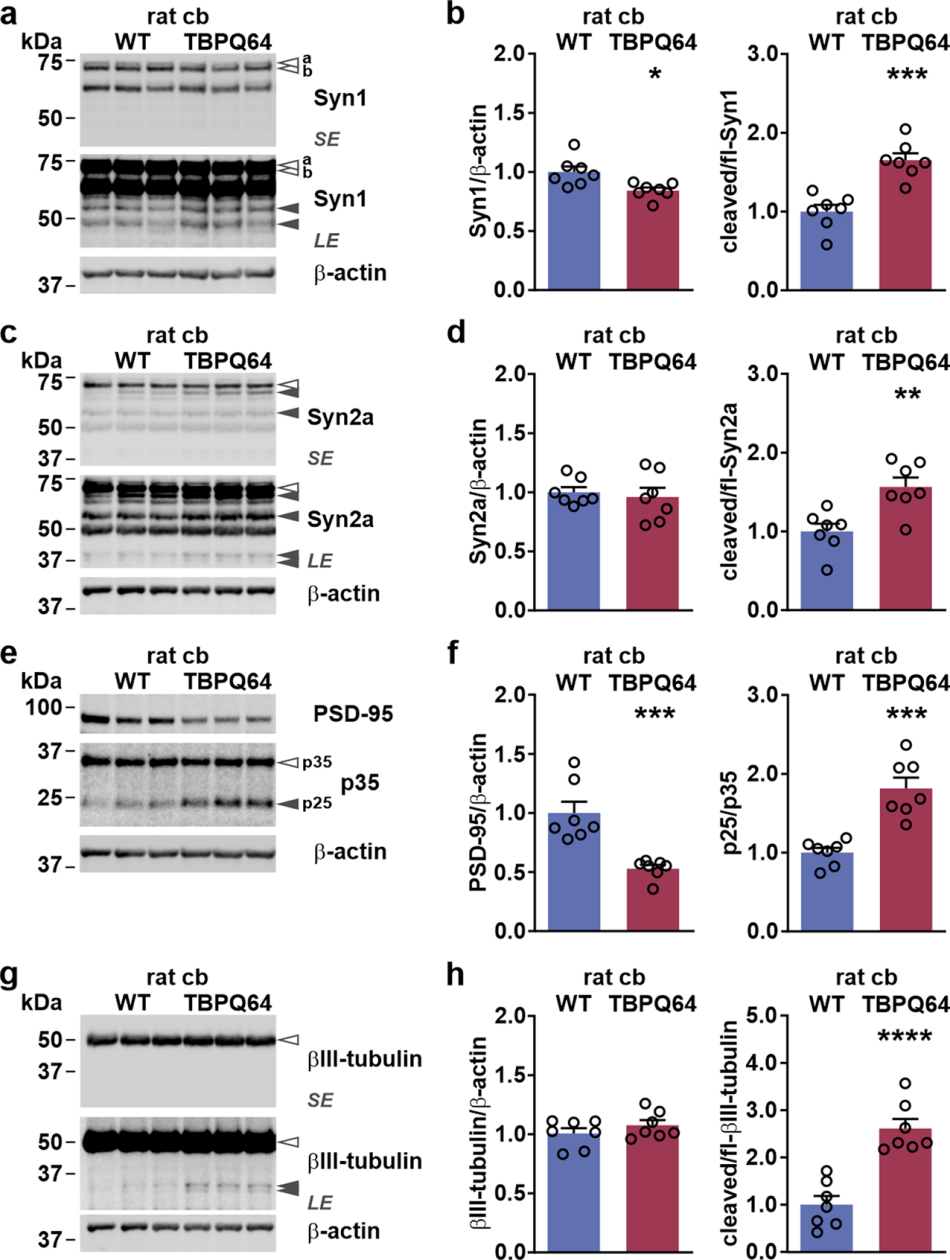
Neuronal proteins are affected by calpain overactivation in TBPQ64 rat cerebellum. **a** Western blot analysis of synapsin-1 (Syn1) in WT and TBPQ64 rat cerebellum (cb). Equal loading was confirmed using β-actin. Empty arrowheads indicate the full-length Syn1 isoforms a and b. Black arrowheads show respective breakdown products. *SE* short exposure; *LE* long exposure. **b** Densitometric analysis shows full-length levels and a cleavage ratio of Syn1. Syn1 levels were normalized to loading control β-actin and cleaved Syn1 to the full-length protein. Obtained values were then normalized to the mean values of WT cerebellum. Bars represent means + SEM. *n* = 7 animals per genotype. *p ≤ 0.05, ***p ≤ 0.001 (Student’s *t*-test). **c** Western blot analysis of synapsin-2a (Syn2a) in WT and TBPQ64 rat cerebellum (cb). Equal loading was confirmed using β-actin. Empty arrowhead indicates full-length Syn2a. Black arrowheads show respective breakdown products. *SE* short exposure; *LE*  long exposure. **d** Densitometric analysis shows full-length levels and cleavage ratio of Sny2a. Syn2a levels were normalized to loading control β-actin and cleaved Syn2a to the full-length protein. Obtained values were then normalized to the mean values of WT cerebellum. Bars represent means + SEM. *n* = 7 animals per genotype. **p ≤ 0.01 (Student’s *t*-test). **e** Western blot analysis of PSD-95 and p35 in WT and TBPQ64 rat cerebellum (cb). Equal loading was confirmed using β-actin. Empty arrowhead indicates p35. Black arrowhead shows p35’s breakdown product p25. **f** Densitometric analysis shows PSD-95 full-length levels and the ratio of p35 to p25 conversion. PSD-95 levels were normalized to loading control β-actin and p25 to the full-length protein p35. Obtained values were then normalized to the mean values of WT cerebellum. Bars represent means + SEM. *n* = 7 animals per genotype. ***p ≤ 0.001 (Student’s *t*-test). **g** Western blot analysis of βIII-tubulin in WT and TBPQ64 rat cerebellum (cb). Equal loading was confirmed using β-actin. Empty arrowhead indicates full-length βIII-tubulin. Black arrowheads show respective breakdown products. *SE* short exposure; *LE* long exposure. **h** Densitometric analysis shows full-length levels and cleavage ratio of βIII-tubulin. βIII-tubulin levels were normalized to loading control β-actin and cleaved βIII-tubulin to the full-length protein. Obtained values were then normalized to the mean values of WT cerebellum. Bars represent means + SEM. *n* = 7 animals per genotype. ****p ≤ 0.0001 (Student’s *t*-test)

In previous studies of our laboratory and other research groups, pathological overactivation of calpains in animal models of various neurodegenerative diseases caused excessive and potentially deleterious fragmentation of neuronal substrates, including synaptic proteins [27, 31, 36, 40, 48]. As we observed elevated calpain activity in the cerebellum of our TBPQ64 rats, we sought to analyze its impact on a selection of known neuronal calpain substrates. For this, we have performed western blot analysis of protein extracts from WT and TBPQ64 rat cerebellum and detected synapsin-1a/b, synapsin-2a and PSD-95, as well as p35. Both synapsins showed a distinct accumulation of fragments accompanied by a reduction of full-length synapsin-1a/b (Fig. 6a, c). In the TBPQ64 cerebellum, fragment levels were approximately 70% higher for synapsin-1a/b and nearly 60% higher for synapsin-2a, whereas full-length levels of synapsin-1a/b were slightly but significantly decreased, and full-length synapsin-2 presented a trend towards lowering when compared to WT samples (Fig. 6b, d). Correspondingly, full-length PSD-95 levels were reduced by half, but we could not detect breakdown products despite showing that calpain-1 readily cleaved this protein (Suppl. Fig. S8a, Supplementary File 1). On the other hand, conversion of p35 to p25 was markedly elevated by almost 90% (Fig. 6e, f), as well as cleavage of the neuron-specific and confirmed calpain substrate βIII-tubulin (Fig. 6g, h; Suppl. Fig. S8b, Supplementary File 1). These observations reveal that the detected calpain overactivation causes strong dysregulations of synaptic proteins and other neuronal proteins in the cerebellum of TBPQ64 rats.

### SCA17 PC12 cells show disturbances of the synaptogenesis and calcium signaling pathways

**Fig. 7:**
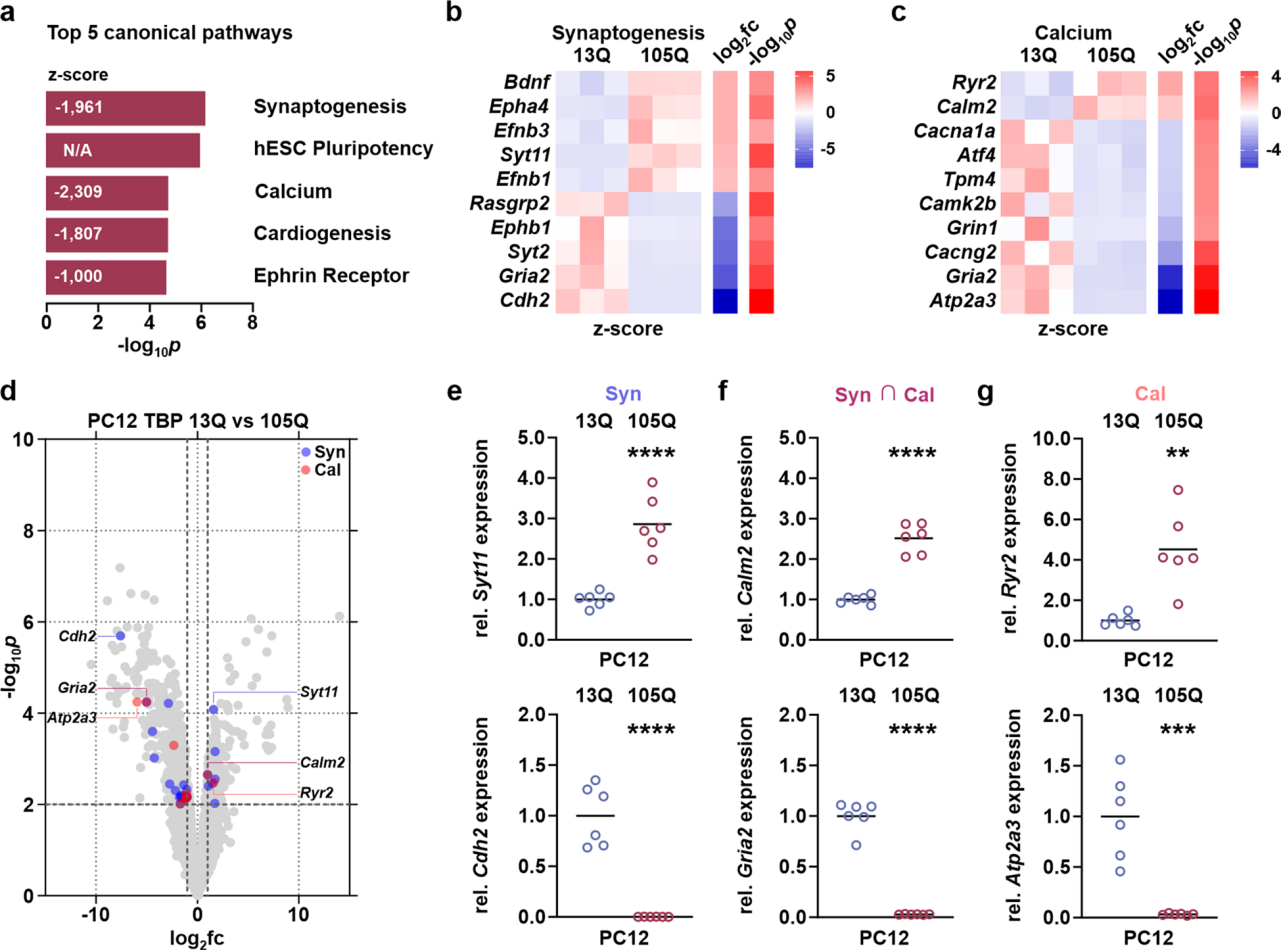
Transcriptome analysis of SCA17 PC12 cells reveals disturbances of synaptogenesis and calcium signaling pathways. **a** Top five dysregulated canonical pathways as obtained by pathway analysis of 3’ RNA sequencing-based transcriptome data of PC12 cells expressing TBP 13Q and 105Q. The graph features both -log_10_*p* values and pathway calculated z-scores. **b** Heatmap of the top 5 up- and downregulated differentially expressed genes (DEGs) within the synaptogenesis pathway. Data is presented as row-wise calculated z-scores, sorted by the log_2_ fold change (log_2_fc). *n* = 3 cell passages per genotype. **c** Heatmap comprising the entire set of DEGs enriched in the calcium signaling pathway. Data is presented as row-wise calculated z-scores, sorted by the log_2_ fold change (log_2_fc). *n* = 3 cell passages per genotype. **d** Volcano plot shows the magnitude of the expression differences (log_2_fc) and their significance (−log_10_*p*), highlighting the most dysregulated DEGs found in synaptogenesis and calcium pathways as well as respective DEGs shared by both pathways. **e, f** Relative (rel.) expression levels of the most dysregulated DEGs within the synaptogenesis (Syn) and calcium (Cal) pathways and shared by both pathways (Syn ∩ Cal) were validated using reverse transcription quantitative real-time PCR. For normalization, reference genes *Actb*, *Pgk1* and *Ubc* were used. Horizontal lines represent means. Obtained values were then normalized to the mean values of TBP 13Q-expressing PC12 cells. *n* = 6 cell passages per genotype. ***p ≤ 0.001, ****p ≤ 0.0001 (Student’s *t*-test)

To scrutinize the connection between polyQ-expanded TBP expression and the overactivation of calpains, we performed a 3’ RNA sequencing of PC12 cells expressing TBP 13Q and 105Q. We detected a total of 1915 differentially expressed genes (DEGs) between both cell lines, of which 795 were up- and 1120 downregulated (Supplementary File 2). Interestingly, genes coding for members of the calpain system including CAST did not show major alterations in their expression levels (Suppl. Fig. S9d, Supplementary File 1), ruling out a direct impact of polyQ-expanded TBP on their transcription. Using the list of identified DEGs, Ingenuity Pathway Analysis revealed synaptogenesis and calcium signaling amongst the top five regulated canonical pathways (Fig. 7a), both showing a significant inhibition. These effects were substantiated by perturbations of mainly downregulated DEGs enriched in both pathways (Supplementary File 2; Fig. 7b, c). Overall, PC12 cells expressing TBP 105Q demonstrated both higher magnitude and significance of DEGs featuring a reduced expression (Fig. 7d). Synaptotagmin 11 (*Syt11*; log_2_fc = 1.60) and cadherin 2 (*Cdh2*; log_2_fc = −7.58) represented the most dysregulated genes in the synaptogenesis pathway (Suppl. Fig. S9a, Supplementary File 1), and ryanodine receptor 2 (*Ryr2*; log_2_fc = 1.61) and sarcoplasmic/endoplasmic reticulum calcium ATPase 3 (*Atp2a3*; log_2_fc = −5.96) were most changed in the calcium signaling pathway (Suppl. Fig. S9c, Supplementary File 1). Both canonical pathways shared several DEGs, including calmodulin 2 (*Calm2*; log_2_fc = 0.61) and glutamate ionotropic receptor AMPA type subunit 2 (*Gria2*; log_2_fc = −5.01) (Suppl. Fig. S9b, Supplementary File 1). We validated the detected expression alterations in these genes by reverse transcription quantitative real-time PCR, which yielded nearly identical fold changes in comparison to our RNA sequencing data (Fig. 7e–g). These results suggest that the observed overactivation of calpains in SCA17 models may be triggered by polyQ-expanded TBP-dependent disturbances of calcium signaling and homeostasis. The transcriptional changes of the synaptogenesis pathway observed in SCA17 PC12 cells, however, may amplify the dysregulation of synaptic proteins as detected in the TBPQ64 rat cerebellum.

### Inhibition of calpains lowers TBP cleavage and aggregation improving cell viability

**Fig. 8:**
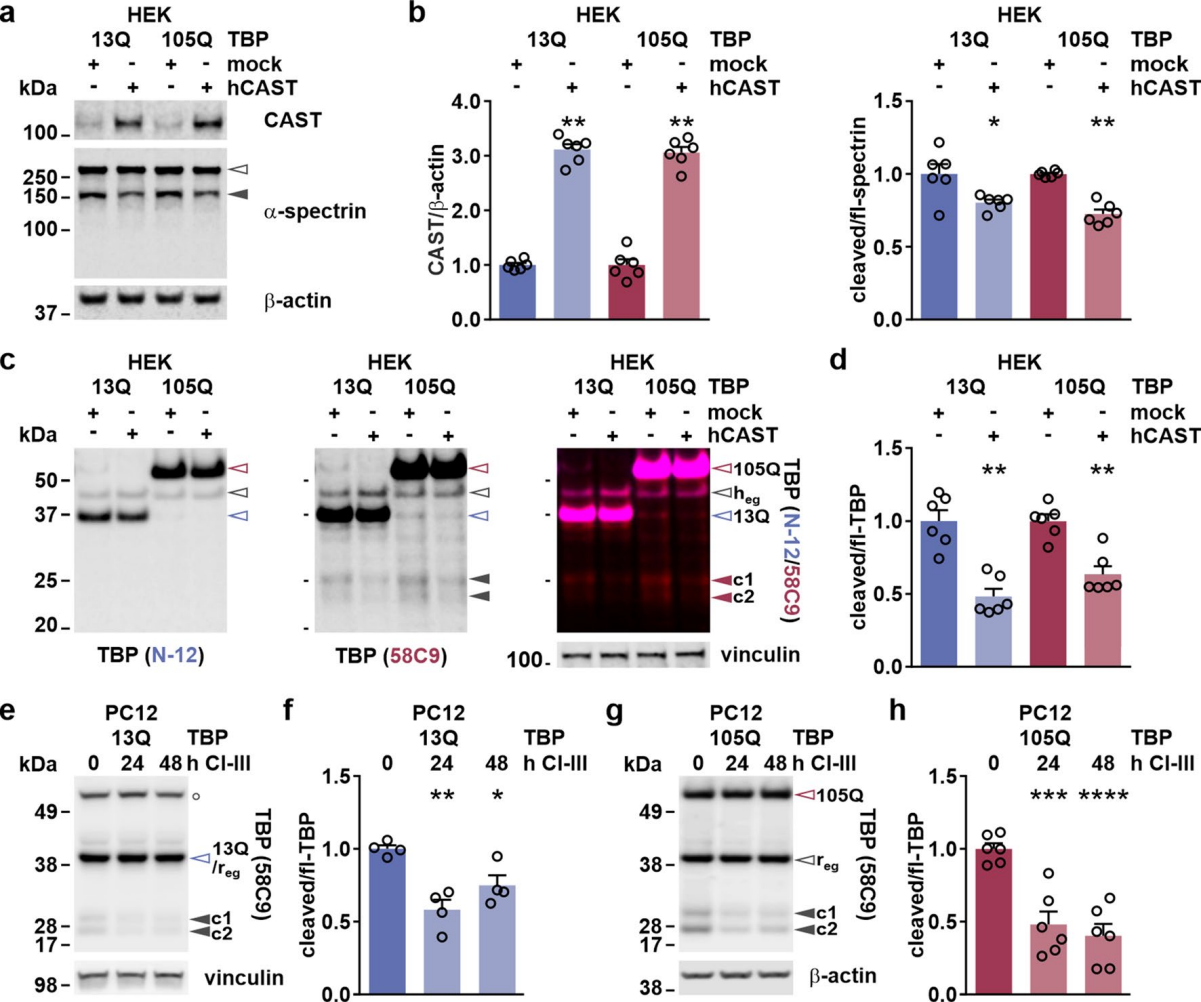
Calpain inhibition by CAST or CI-III lowers TBP cleavage in cell models of SCA17. **a** HEK 293T (HEK) cells co-expressing TBP 13Q or 105Q together with human CAST (hCAST) or a mock vector for 72 h were analyzed by western blotting and immunodetection using CAST and α-spectrin antibodies. Equal loading was confirmed using β-actin. Empty arrowhead indicates full-length α-spectrin, and the black arrowhead its respective breakdown product. **b** Densitometric analysis shows the overexpression of CAST and α-spectrin cleavage as a marker of calpain inhibition. CAST levels were normalized to loading control β-actin and cleaved α-spectrin to the full-length protein. Obtained values were then normalized to the respective TBP genotype control 13Q or 105Q. Bars represent means + SEM. *n* = 6 repeated transfections at different time points. *p ≤ 0.5, **p ≤ 0.01 (Student’s *t*-test). **c** Western blot analysis of TBP fragmentation was performed using the TBP antibodies N-12 and 58C9. Equal loading was confirmed using vinculin. Empty arrowheads indicate full-length TBP: red-rimmed = 105Q, blue-rimmed = 13Q, black-rimmed = endogenous human TBP (h_eg_). Red or black arrowheads indicate C-terminal TBP fragments (c1/c2). **d** Densitometric analysis shows effects on TBP fragmentation upon CAST overexpression. Cleaved TBP was normalized to the full-length protein and then to the respective TBP genotype control 13Q or 105Q. Bars represent means + SEM. *n* = 6 repeated transfections at different time points. **p ≤ 0.01 (Student’s *t*-test). **e, g** PC12 cells expressing TBP with 13Q or 105Q were treated with the calpain inhibitor CI-III for 24 h or 48 h. Samples were analyzed by western blotting and immunodetection with the TBP 58C9 antibody. Equal loading was confirmed using β-actin. Empty arrowheads indicate full-length proteins: red-rimmed = TBP 105Q, blue-rimmed = TBP 13Q and endogenous rat TBP (r_eg_), black-rimmed = endogenous rat TBP (r_eg_). Black arrowheads indicate the C-terminal TBP fragments (c1/c2). White circle (○) indicates an unspecific band. **f, h** Densitometric analysis shows effects on TBP fragmentation upon CI-III treatment for 24 h or 48 h. Cleaved TBP was normalized to the full-length protein and then to the respective TBP genotype control 13Q or 105Q. Bars represent means + SEM. *n* = 4 (13Q) or 6 (105Q) repeated transfections/treatments at different time points. *p ≤ 0.05, **p ≤ 0.01, ***p ≤ 0.001, ****p ≤ 0.0001 (one-way ANOVA with Dunnett’s post hoc test)

Calpain inhibition by overexpression of CAST or pharmacological means has been proven effective in reducing levels of toxic fragments and aggregates of mutant proteins, thereby attenuating the overall pathology in models of neurodegenerative diseases including HD, MJD and PD [28, 34–36, 49, 50]. Therefore, we tested both genetic and pharmacological approaches on our SCA17 cell models for evaluating their impact on TBP cleavage and aggregation as well as cell viability. In our first attempt, we overexpressed human CAST in HEK 293T cells together with TBP 13Q or 105Q for 72 h and analyzed the obtained protein extracts via western blotting. We achieved a threefold increase of CAST resulting in an approximately 30% reduction of α-spectrin cleavage, which was detected as a marker of overall calpain activity (Fig. 8a, b). By immunodetection of TBP with antibodies N-12 and 58C9, we observed a significant 40–50% lowering of the C-terminal fragments c1 and c2 upon CAST overexpression (Fig. 8c, d), again confirming the calpain-dependent origin of these TBP breakdown products. To test how this reduction manifests on the subcellular level, we performed a cytoplasmic-nuclear fractionation of HEK 293T co-expressing CAST and myc-TBP 38Q or 64Q (Suppl. Fig. S10a, Supplementary File 1). Quantitative analysis reproduced the previous findings of the mainly cytoplasmic localization of the fragments c1 and c2, showing that levels of both breakdown products were significantly lowered in the cytoplasm but not in nuclear fraction upon CAST overexpression (Suppl. Fig. S10b, Supplementary File 1). To reproduce the findings using a pharmacological strategy in an additional cell model, we treated PC12 cells expressing TBP 13Q and 105Q with CI-III for 24 h or 48 h. Western blot analysis using the 58C9 antibody demonstrated a markedly reduced fragmentation of both TBP 13Q (Fig. 8e, f) and 105Q (Fig. 8g, h), which appeared to be slightly more effective for the polyQ-expanded TBP (Fig. 8f, h).

**Fig. 9:**
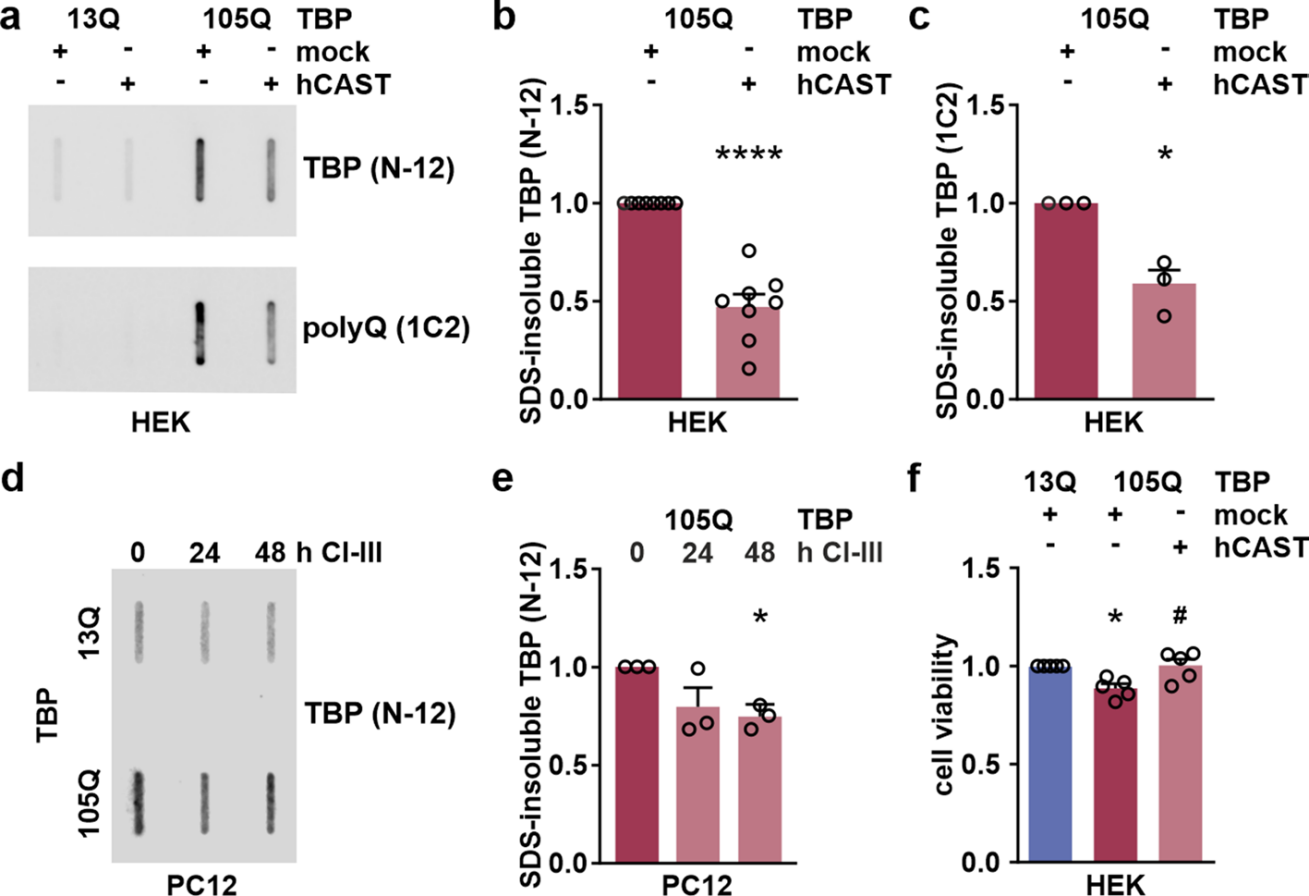
Calpain inhibition reduces TBP aggregation and rescues cell viability of SCA17 cell lines. **a** HEK 293T (HEK) cells co-expressing TBP 13Q or 105Q together with human CAST (hCAST) or a mock vector for 72 h were analyzed using filter retardation assays and immunodetection with TBP antibody N-12 or polyQ-specific antibody 1C2. **b, c** Densitometric analysis shows levels of SDS-insoluble forms of TBP in HEK 293T cells. Levels were normalized to the respective control (mock vector co-transfected cells expressing TBP 105Q) within each sample set. Bars represent means + SEM. *n* = 8 (**b**) or *n* = 3 (**c**) repeated transfections at different time points. *p ≤ 0.05, ***p ≤ 0.001 (one-sample *t*-test). **d** PC12 cells expressing TBP with 13Q or 105Q were treated with CI-III for 24 h or 48 h. DMSO was used as vehicle control. Samples were analyzed using filter retardation assays and immunodetection with TBP antibody N-12. **e** Densitometric analysis shows levels of SDS-insoluble forms of TBP in PC12 cells after 24 h and 48 h treatment with CI-III. Levels were normalized to the respective control (DMSO-treated cells expressing TBP 105Q) within each sample set. Bars represent means + SEM. *n* = 3 passages per genotype and repeated treatments. *p ≤ 0.05 (one-sample *t*-test). **f** Cell viability of HEK 293T cells co-expressing TBP 13Q or 105Q together with human CAST (hCAST) or a mock vector for 72 h was assessed using the PrestoBlue assay. Signals were normalized to respective control (mock vector co-transfected cells expressing wild-type TBP 13Q) for each measurement. *compared to 13Q/mock; ^#^compared to 105Q/mock. *n* = 5 repeated transfections at different time points. *^/#^*p* ≤ 0.05 (one-sample or Student’s *t*-test)

As fragment levels for expanded- and unexpanded TBP were modulated likewise by the calpain inhibition, we then assessed its repercussion on TBP aggregate formation and cell viability. For aggregate analysis, filter retardation assays and denaturing detergent agarose gel electrophoresis (DD-AGE) of protein extracts from HEK 293T expressing TBP 105Q and CAST (Fig. 9a–c; Suppl. Fig. S11, Supplementary File 1) as well as from PC12 cells expressing TBP 105Q treated with CI-III for 24 h or 48 h (Fig. 9d, e) were performed, using the aggregate-detecting TBP antibody N-12. Results from both cell lines showed a significant decrease in SDS-insoluble species of polyQ-expanded TBP. In HEK 293T cells, this effect was accompanied by a significant rescue of the impaired viability in cells co-expressing TBP 105Q and CAST, as detected by a resazurin-based assay (Fig. 9f).

Taken together, we demonstrated that calpain inhibition ameliorates TBP cleavage and aggregation, and rescues impaired cell viability, which all represent typical hallmarks of SCA17’s molecular pathology.

## Discussion

We here demonstrate the involvement of calpains as new players in the molecular pathogenesis of SCA17. Based on our investigations in SCA17 cell and animal models, we showed that, first, the disease protein TBP is cleaved by calpains; second, that calpains are overactivated and compromise vital neuronal proteins; and third, that calpain inhibition by genetic and pharmacological approaches ameliorates molecular disease hallmarks. Moreover, our transcriptome analysis of SCA17 cells revealed dysregulations of the synaptogenesis and calcium signaling pathways, the latter suggesting being the trigger for calpain overactivation.

The occurrence of truncated, more toxic forms of disease proteins as a pathological key factor has been associated with multiple neurodegenerative diseases, such as AD or polyQ disorders, which gave rise to the toxic fragment hypothesis [17, 18, 51]. Fragments of polyQ-expanded TBP could also be crucial for disease development and have been found in SCA17 mouse and AD patient brains [14, 15]. However, earlier studies have failed to link the presence of TBP fragments to a proteolytic event such as caspase-mediated cleavage [19]. Moreover, an alternative splicing event giving rise to TBP species consisting of an N-terminal portion was presented as an explanation for accumulating fragments in AD brains [16]. In our study, we revealed that wild-type and polyQ-expanded TBP are efficiently fragmented by calpains, as demonstrated using in vitro approaches with purified calpain-1 and -2 as well as overactivation of endogenous calpains in living cells. This processing explained the occurrence of two specific and predominant C-terminal fragments of the disease protein, called here c1 and c2, both in SCA17 cells and in the cerebellum of TBPQ64 rats. Together with in silico predictions, analysis of the obtained fragmentation pattern could narrow down the location of calpain cleavage sites of c1 and c2 to a region C-terminal of the polyQ-stretch, flanking the epitope of the antibody D5G7Y at Ala110. A precise mapping of these cleavage sites, followed by a further analysis of the originating fragments and their disease-relevant properties, might give important insight into the role of these fragments in the molecular pathogenesis of SCA17, as previously conducted for huntingtin in HD and of ataxin-3 in MJD [26, 50].

To follow up on some of the characteristics of fragments c1 and c2, we performed cytoplasmic-nuclear fractionation of TBP-transfected HEK 293T cells. Importantly, the detrimental mislocalization of disease proteins or fragments has been reported for several neurodegenerative disorders, including MJD and HD [52–55]. Although full-length TBP showed, as expected, a mainly nuclear localization, fragments c1 and c2 were intriguingly enriched in the cytoplasmic fraction. Fractionation and microscopy analysis of members of the calpain system revealed their predominantly cytoplasmic localization, giving rise to the questions where calpain-mediated cleavage of TBP takes place and how the TBP’s DNA-binding domain-containing C-terminal portions may mislocalize. The observed minor spatial overlap based on the small nuclear fraction of calpains or the cytoplasmic share of TBP might indicate areas of the proteins’ interaction. Interestingly, subcellular localization signals within TBP have been only predicted, without directly characterizing a specific nuclear localization signal (NLS) or even nuclear export signal (NES) [14, 56]. The cytoplasmic mislocalization of N-terminal fragments of polyQ-expanded TBP was however attributed to the separation from a putative C-terminal NLS [14], whose existence would contradict the cytoplasmic retention of fragments c1 and c2 observed in our work. Interestingly, our in silico predictions hinted at a potential NES at Leu275 of TBP’s C-terminus and thus within the fragments’ boundaries, which may explain why both breakdown products are mainly present in the cytoplasm. Still, further investigations on fragments c1 and c2 are demanded to clarify the cause of their localization and to evaluate their potential biological consequences. This might be of particular relevance, as even non-polyQ fragments of disease proteins ataxin-3 and huntingtin were demonstrated to contribute to the pathogenesis of the respective diseases MJD and HD [57, 58].

Besides their involvement in disease protein cleavage, calpains have been connected to additional neurodegenerative processes, which feature their pathological overactivation and the excessive fragmentation of neuronal substrates [59–61]. In our laboratory’s studies on HD, PD and MJD, it was demonstrated that calpain overactivation is accompanied by increased proteolysis of synaptic proteins in respective mouse models [27, 36, 40]. When analyzing markers of global calpain activation in a PC12 cell model of SCA17, we observed the lowering of CAST levels and an increase of α-spectrin cleavage, indicative of a general overactivation of these proteases. We reproduced these findings in cerebellum of 10 months old of TBPQ64 rats, including an elevated TBP cleavage at baseline. Importantly, in brain areas exhibiting low expression of polyQ-expanded TBP, calpain overactivation was not detectable, emphasizing that the presence of the disease protein triggers this dysregulation. The increased activity of calpains induces the cleavage of synapsin-1, synapsin-2, βIII-tubulin and p35, as well as a lowering of PSD-95 levels in the TBPQ64 rat cerebellum. Neuronal dysfunction and death, which have been connected to elevated fragmentation or lower occurrence of these proteins [48, 62, 63], might explain some of the neuropathological and behavioral characteristics described for our SCA17 rat model [39, 64]. Nonetheless, future investigations should verify the calpain system overactivation and neuronal protein cleavage in additional models, such as TBP-71Q mice [13], or especially in SCA17 patient-derived cells and tissues, to further corroborate our findings.

The actual reason for the observed overactivation of the calpain system demanded clarification. By performing RNA sequencing-based transcriptome analysis and quantitative PCR we detected negative dysregulations of the synaptogenesis and calcium signaling pathways in PC12 cells expressing polyQ-expanded TBP. Interestingly, genes of the calpain system did not exhibit major changes, suggesting that disturbances in calcium homeostasis such as the upregulation of ryanodine receptor 2 or downregulation of sarcoplasmic/endoplasmic reticulum calcium ATPase 3 might trigger a calpain-activating increase of cytoplasmic calcium concentrations. Preventing leakage or improving the uptake of calcium into the endoplasmic reticulum constitutes, therefore, a potential pharmacological target for counteracting pathological calpain overactivation in SCA17 [65, 66]. Correspondingly, a recent study, which included transcriptome analysis of SCA17 knock-in mice, also demonstrated the pathological impact of dysfunctional calcium signaling in SCA17 cerebellum [67].

The observed and potentially detrimental calpain-mediated cleavage of polyQ-expanded TBP and the overactivation of calpains constitute auspicious therapeutic targets for treating SCA17. Engaging this system with pharmacological tools such as specific calpain inhibitors or compounds targeting activating pathways as well as genetic strategies, like overexpressing CAST or ablating calpain isoforms, showed phenotype-attenuating effects in models of neurodegenerative diseases [27–29, 33–38]. By overexpression of CAST and administration of the inhibitor CI-III, we were able to lower disease protein cleavage and aggregation as well as improve viability in mutant TBP-expressing HEK 293T and PC12 cells. These beneficial effects might be a direct consequence of the reduced TBP cleavage. On the other hand, calpains have been shown to negatively regulate autophagy, and their inhibition led to the removal of disease protein species by improving their autophagic turnover [37, 68]. To evaluate calpain inhibition as an applicable treatment for SCA17, translation of these inhibitory approaches into an in vivo model is an inevitable next step. However, it must be taken into consideration that calpains execute important cellular functions. Non-optimized interventions on these proteases, e.g., targeting a vital isoform or application of unspecific inhibitors, might lead to undesirable and deleterious side-effects [59, 69].

We demonstrated that TBP is cleaved by calpains, leading to the generation of C-terminal fragments that contain the DNA-binding core and mislocalize to the cytoplasm. Moreover, calpains were pathologically overactivated in SCA17 cells and TBPQ64 rat cerebellum, provoking disturbances of multiple neuronal proteins. In SCA17 cells, we detected transcriptional dysregulations of synaptogenesis and calcium signaling pathways, potentially triggering calpain overactivation. Coherently, calpain inhibition attenuated both TBP fragmentation and aggregation, thereby rescuing the compromised cell viability. Hence, SCA17 joins the ranks of a large group of neurodegenerative disorders which share calpain overactivation as a unifying, therapeutically targetable disease mechanism.

## Supplementary Information

Below is the link to the electronic supplementary material.

Supplementary file1 (PDF 923 KB)

Supplementary file2 (XLSX 1891 KB)

## Data Availability

The data generated or analyzed during this study are included in this published article and its supplementary files.

## Abbreviations

AD: Alzheimer disease
ALS: Amyotrophic lateral sclerosis
CAPN1: Calpain-1
CAST: Calpastatin
hCAST: Human CAST
cb: Cerebellum
CB CCA: Cell-based calpain cleavage assay
CI-III: Calpain inhibitor III
cx: Cortex
HD: Huntington disease
HDL4: HD-like 4
IV CCA: In vitro calpain cleavage assay
MJD: Machado–Joseph disease
NES: Nuclear export signal
NLS: Nuclear localization signal
PD: Parkinson disease
polyQ: Polyglutamine
qPCR: Quantitative real-time PCR
SCA17: Spinocerebellar ataxia type 17
SMA: Spinal muscular atrophy
st: Striatum
TBP: TATA box-binding protein
TBPQ64 rat: SCA17 transgenic rat model expressing human TBP with 64 glutamines from a yeast artificial chromosome under the control of the murine prion promoter
WT: Wild-type

## Code availability

The codes used for analyzing the data are available from the corresponding author on reasonable request.
