# Supplementary material for: Calpains as novel players in the molecular pathogenesis of spinocerebellar ataxia type 17
Source: Cell Mol Life Sci. 2022 Apr 28;79(5):262. doi: 10.1007/s00018-022-04274-6 (PMC9050766; doi:10.1007/s00018-022-04274-6)
Supplement: Supplementary file 1 — Supplementary file1 (PDF 923 KB) [file 18_2022_4274_MOESM1_ESM.pdf]

Supplementary File 1

Calpains as novel players in the molecular pathogenesis of spinocerebellar ataxia type 17

Jonasz Jeremiasz Weber[1,2,#], Stefanie Cari Anger[2,#], Priscila Pereira Sena[2,3], Rana Dilara Incebacak Eltemur[1,2], Chrisovalantou Huridou[2], Florian Fath[1,2], Caspar Gross[2,4], Nicolas Casadei[2,4], Olaf Riess[2,4], Huu Phuc Nguyen[1,*]

[1] Department of Human Genetics, Ruhr University Bochum, 44801 Bochum, Germany

[2] Institute of Medical Genetics and Applied Genomics, University of Tübingen, 72076 Tübingen, Germany

[3] Graduate School of Cellular Neuroscience, University of Tübingen, 72074 Tübingen, Germany

[4] NGS Competence Center Tübingen, 72076 Tübingen, Germany

[#]These authors contributed equally.

***Correspondence should be addressed to:**

Prof. Dr. med. Huu Phuc Nguyen

Department of Human Genetics, Ruhr-University Bochum, Universitätsstraße 150, 44801 Bochum, Germany

Phone: +49 234 32 23839

Fax: +49 234 32 14196

Email: huu.nguyen-r7w@rub.de

Supplementary Methods

Denaturing detergent agarose gel electrophoresis (DD-AGE)

To analyze higher molecular species of TBP, denaturing detergent-agarose gel electrophoresis (DD-AGE) was performed according to the previously described protocol (1), with following modifications. 25 µg of cell homogenate were mixed in a ratio 3:1 with 4x DDAGE sample buffer (2x TAE, 50% (v/v) glycerol, 8% (w/v) LDS, and 0.1% (w/v) Orange G) supplemented with 100 mM DTT, and subsequently heat-denatured at 95°C for 5 min. Separation of samples occurred electrophoretically on a 1% TAE agarose gel with 0.1% (w/v) SDS using a PerfectBlue™ Gelsystem Mini S (PEQLAB) at 45 V for approx. 2 h. For detecting higher molecular species of TBP, primary antibody rabbit anti-TBP (1:1000; N-12, sc-204, Santa Cruz Biotechnology) was used. Huntingtin was detected as loading control using primary antibody mouse anti-huntingtin antibody (1:1000; clone 1HU-4C8, MAB2166, Merck Millipore).

Supplementary References

1. Weber JJ, Golla M, Guaitoli G, Wanichawan P, Hayer SN, Hauser S, et al. A combinatorial approach to identify calpain cleavage sites in the Machado-Joseph disease protein ataxin-3. Brain. 2017 May 1;140(5):1280–99.

Supplementary Tables

Suppl. Table S1. Primary antibodies for western blot analysis.

Antibody	Host species	Dilution	Clone no., catalogue no.	Provider
anti-β-actin	mouse	1:10,000	clone AC-15, A5441	Sigma-Aldrich
anti-calpain-1	rabbit	1:1000	ab39170	Abcam
anti-calpain-2	rabbit	1:1000	ab39168	Abcam
anti-calpastatin	rabbit	1:1000	#4146	Cell Signaling
anti-GAPDH	mouse	1:2500	clone 0411, sc-47724	Santa Cruz Biotech.
anti-lamin A/C	rabbit	1:2500	sc-20681	Santa Cruz Biotech.
anti-c-myc	mouse	1:200	clone 9E10, sc-40	Santa Cruz Biotech.
anti-p35/p25	rabbit	1:1000	clone C64B10, #2680	Cell Signaling
anti-polyglutamine/TBP	mouse	1:1000	5TF1-1C2, MAB1574	Merck Millipore
anti-PSD-95	goat	1:1000	ab12093	Abcam
anti-α-spectrin	mouse	1:1000	clone AA6, MAB1622	Merck Millipore
anti-synapsin-1a/b	rabbit	1:500	sc-20780	Santa Cruz Biotech.
anti-synapsin-2a	rabbit	1:500	sc-25538	Santa Cruz Biotech.
anti-TBP	mouse	1:1000	clone 58C9, T1827	Sigma-Aldrich
anti-TBP	rabbit	1:500	#8515	Cell Signaling
anti-TBP	rabbit	1:500	clone D5G7Y, #12578	Cell Signaling
anti-TBP	rabbit	1:500	N-12, sc-204	Santa Cruz Biotech.
anti-α-tubulin	mouse	1:5000	clone DM1A, CP06	Merck Millipore
anti-βIII-tubulin	mouse	1:2500	clone TuJ-1, MAB1195	R&D Systems
anti-vinculin	rabbit	1:1000	clone E1E9V, #13901	Cell Signaling

Suppl. Table S2. Primers used for reverse transcription quantitative PCRs to validate differentially expressed genes in rat PC12 cells.

Gene	Orientation	Primer sequence 5' – 3'
Atp2a3	forward	CTATTTGGCTATTGGAGTGTAC
	reverse	CTGATGGAAGGTGACTTGTGG
Calm2	forward	GTGGATGCTGATGGCAATGG
	reverse	CTCGTATCTCCTCCTCGCTG
Cdh2	forward	GGCAATCAAGTGGAGAACCC
	reverse	CATCACATACGTCCCAGGCTT
Gria2	forward	CATTTCGGGTAGGGATGGTTC
	reverse	GGAGCAGAAAGCATTGGTGAC
Ryr2	forward	GACATGCTGACGTGTTACATG
	reverse	ACTCATCTCCTGCTGGGTCT
Syt11	forward	GCCCCAGCTTTGATGTGTCA
	reverse	TCTTGTGCTTCTTCTCCGCC
Actb	forward	CAACTGGGACGATATGGAGAAG
	reverse	TCTGGGTCATCTTTTCACGG
Pgk1	forward	GTTGTGCTTATGAGCCACCTG
	reverse	AAGAACAGAACATCCTTGCCC
Ubc	forward	ACTCGTACCTTTCTCACCACA
	reverse	CCTCCCCATCAAACCCAAGA

Suppl. Table S3. Best hits for predicted calpain cleavage sites in TBP using the GPS-CCD tool.

Amino acid positions (Pos.) with a calpain cleavage likelihood score above the software-determined maximal threshold of 0.654 and their respective sequences are listed from the N-terminus to the C-terminus of TBP reference isoform 1 (UniProt identifier: P20226-1). Predicted position of the cleavage site is indicated by a vertical bar (|). High scores for the polyglutamine stretch (in red) were considered as artifacts of the *in silico* prediction.

Pos.	Sequence	Score	Pos.	Sequence	Score
N5	*****MDQNN|SLPP	0.946	Q94	QQQQQQQQQQ|QAVA	1.323
A15	SLPPYAQGLA|SPQG	0.712	Q95	QQQQQQQQQQ|AVAA	1.560
G19	YAQGLASPQG|AMTP	0.750	A96	QQQQQQQQQA|VAAA	1.114
E53	QNTNSLSILE|EQQR	0.696	V97	QQQQQQQQAV|AAAA	1.253
Q56	NSLSILEEQQ|RQQQ	0.747	A98	QQQQQQQAVA|AAAV	1.701
Q60	ILEEQQRQQQ|QQQQ	1.174	A99	QQQQQQAVAA|AAVQ	1.022
Q61	LEEQQRQQQQ|QQQQ	1.457	A100	QQQQQAVAAA|AVQQ	1.220
Q62	EEQQRQQQQQ|QQQQ	1.622	A101	QQQQAVAAAA|VQQS	1.125
Q63	EQQRQQQQQQ|QQQQ	1.688	V102	QQQAVAAAAV|QQST	0.875
Q64	QQRQQQQQQQ|QQQQ	1.804	Q103	QQAVAAAAVQ|QSTS	1.302
Q65	QRQQQQQQQQ|QQQQ	1.769	Q104	QAVAAAAVQQ|STSQ	1.122
Q66	RQQQQQQQQQ|QQQQ	1.565	S105	AVAAAAVQQS|TSQQ	1.160
Q67	QQQQQQQQQQ|QQQQ	1.840	T106	VAAAAVQQST|SQQA	1.438
Q68	QQQQQQQQQQ|QQQQ	1.840	S107	AAAAVQQSTS|QQAT	1.288
Q69	QQQQQQQQQQ|QQQQ	1.840	Q108	AAAVQQSTSQ|QATQ	0.976
Q70	QQQQQQQQQQ|QQQQ	1.840	Q109	AAVQQSTSQQ|ATQG	1.171
Q71	QQQQQQQQQQ|QQQQ	1.840	A110	AVQQSTSQQA|TQGT	1.052
Q72	QQQQQQQQQQ|QQQQ	1.840	T111	VQQSTSQQAT|QGTS	1.391
Q73	QQQQQQQQQQ|QQQQ	1.840	Q112	QQSTSQQATQ|GTSG	1.334
Q74	QQQQQQQQQQ|QQQQ	1.840	G113	QSTSQQATQG|TSGQ	1.220
Q75	QQQQQQQQQQ|QQQQ	1.840	T114	STSQQATQGT|SGQA	0.872
Q76	QQQQQQQQQQ|QQQQ	1.840	S115	TSQQATQGTS|GQAP	1.402
Q77	QQQQQQQQQQ|QQQQ	1.840	G116	SQQATQGTSG|QAPQ	1.160
Q78	QQQQQQQQQQ|QQQQ	1.840	Q117	QQATQGTSGQ|APQL	0.899
Q79	QQQQQQQQQQ|QQQQ	1.840	T128	PQLFHSQTLT|TAPL	0.693
Q80	QQQQQQQQQQ|QQQQ	1.840	T129	QLFHSQTLTT|APLP	0.875
Q81	QQQQQQQQQQ|QQQQ	1.840	P133	SQTLTTAPLP|GTTP	0.785
Q82	QQQQQQQQQQ|QQQQ	1.840	G134	QTLTTAPLPG|TTPL	0.717
Q83	QQQQQQQQQQ|QQQQ	1.840	Y139	APLPGTTPLY|PSPM	0.668
Q84	QQQQQQQQQQ|QQQQ	1.840	A155	MTPITPATPA|SESS	0.715
Q85	QQQQQQQQQQ|QQQQ	1.840	S159	TPATPASESS|GIVP	0.783
Q86	QQQQQQQQQQ|QQQQ	1.840	G160	PATPASESSG|IVPQ	0.997
Q87	QQQQQQQQQQ|QQQQ	1.840	Q166	ESSGIVPQLQ|NIVS	0.793
Q88	QQQQQQQQQQ|QQQQ	1.840	F214	REPRTTALIF|SSGK	0.793
Q89	QQQQQQQQQQ|QQQQ	1.840	T222	IFSSGKMVCT|GAKS	0.728
Q90	QQQQQQQQQQ|QQQQ	1.840	A233	AKSEEQSRLA|ARKY	1.019
Q91	QQQQQQQQQQ|QQQQ	1.840	A238	QSRLAARKYA|RVVQ	0.693
Q92	QQQQQQQQQQ|QQQA	1.750	G245	KYARVVQKLG|FPAK	0.728
Q93	QQQQQQQQQQ|QQAV	1.780	T313	IFVSGKVVLT|GAKV	0.709

Suppl. Table S4. Predicted NES in TBP using the NetNES tool.

NES scores above the default cut-off value of 0.5 and their sequence information are listed for a region of interest (amino acid positions 267-283) at the C-terminus of TBP (reference isoform 1; UniProt identifier: P20226-1). Pos. = amino acid position, ANN = artificial neural network, HMM = hidden Markov model. Predicted NES at L275 is highlighted in blue.

Pos.	ANN	HMM	NES score	Predicted
P267	0.075	0.044	0.000	-
I268	0.090	0.380	0.084	-
R269	0.069	0.380	0.084	-
L270	0.269	0.380	0.115	-
E271	0.094	0.378	0.117	-
G272	0.121	0.378	0.121	-
L273	0.102	0.378	0.127	-
V274	0.074	0.376	0.122	-
L275	0.754	0.376	0.674	Yes
T276	0.078	0.000	0.000	-
H277	0.179	0.000	0.000	-
Q278	0.098	0.000	0.000	-
Q279	0.099	0.000	0.000	-
F280	0.110	0.000	0.000	-
S281	0.075	0.000	0.000	-
S282	0.077	0.000	0.000	-
Y283	0.096	0.000	0.000	-

Supplementary Figures and Figure Legends

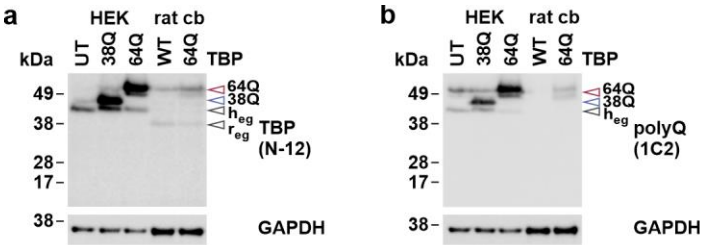

Suppl. Fig. S1 Immunodetection of TBP on PVDF membranes using N-terminally binding antibodies. **a and b.** Protein extracts of untransfected (UT) and myc-TBP 38Q or 64Q-transfected HEK 293T (HEK) cells, and of wild-type (WT) or TBPQ64 (64Q) rat cerebellum (cb) were assayed by western blotting using PVDF membranes. TBP was detected by the TBP-specific antibody N-12 and the polyQ-specific antibody 1C2. GAPDH served as a loading control. Empty arrowheads indicate full-length TBP: red-rimmed = 64Q, blue-rimmed = 38Q, black-rimmed = endogenous human/ rat TBP (h_{eg}/ r_{eg})

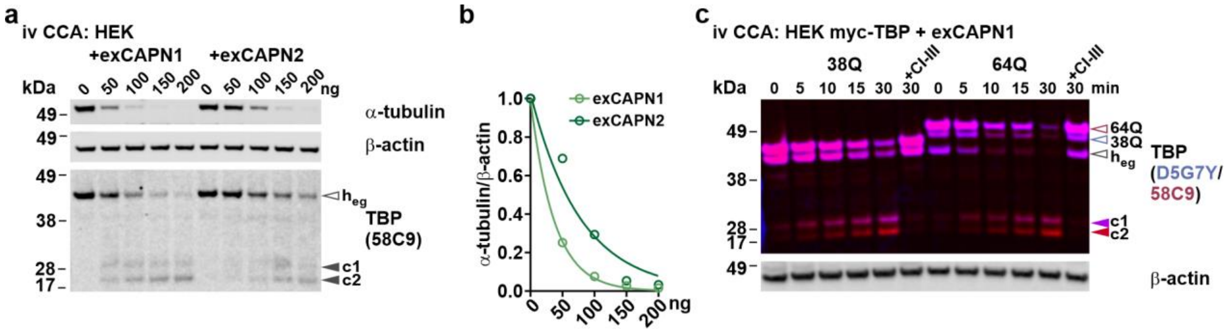

Suppl. Fig. S2 *In vitro* cleavage assay establishment and double labelling of fragment c1 with antibodies 58C9 and D5G7Y. **a.** For assessing comparable amounts of both proteases within *in vitro* calpain cleavage assays (iv CCA) in terms of reaction rates, 25 µg of protein extract from untransfected HEK 293T (HEK) cells was incubated with 0 – 200 ng of purified calpain-1 (exCAPN1) or calpain-2 (exCAPN2) for 15 min. After western blotting, α-tubulin was detected as a reference substrate for the reaction rate. Equal loading was confirmed using β-actin. To assess effects on TBP cleavage, antibody 58C9 was applied. Black-rimmed arrowhead marks endogenous human TBP (h_{eg}). Black arrowheads indicate C-terminal TBP fragments (c1/ c2). **b.** Densitometric analysis shows reduction of α-tubulin levels when incubated with exCAPN1 (light green circles) or exCAPN2 (dark green circles). α-tubulin levels were first normalized to loading control β-actin and then to respective levels of sample without addition of exogenous calpain. Based on nonlinear regression analyses, 100 ng of exCAPN1 and 200 ng of exCAPN2 were applied in subsequent iv CCAs. **c.** Protein extracts of myc-TBP 38Q or 64Q-transfected HEK 293T cells were incubated with purified calpain-1 (exCAPN1) for up to 30 min. Calpain inhibitor III (CI-III) controls were performed to validate specificity of the reaction. Samples were analyzed by western blotting and double labelling using the TBP-specific antibodies D5G7Y and 58C9. Equal loading was confirmed using β-actin. Empty arrowheads indicate full-length TBP: red-rimmed = 64Q, blue-rimmed = 38Q, black-rimmed = endogenous human TBP (h_{eg}). Purple arrowhead indicates C-terminal TBP fragment c1 detected by both TBP antibodies, red arrowhead marks C-terminal fragment c2 labelled by antibody 58C9 only

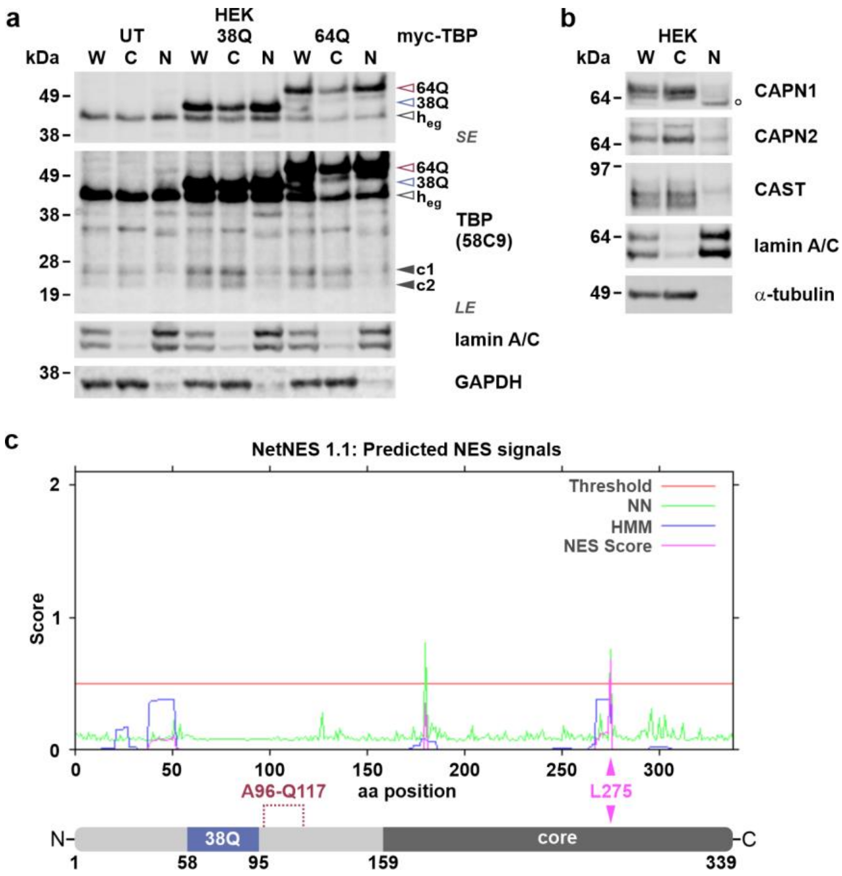

Suppl. Fig. S3 C-terminal fragments of endogenous and overexpressed TBP as well as calpain system members are enriched in the cytoplasm. **a.** Untransfected (UT) or myc-TBP 38Q or 64Q-transfected HEK 293T (HEK) cells were subjected to cytoplasmic-nuclear fractionation followed by western blotting. Full-length TBP and its C-terminal fragments c1 and c2 were detected using antibody 58C9. The nuclear proteins lamin A and C, and cytoplasmic protein GAPDH served as fraction purity controls. W = whole cell, C = cytoplasmic fraction, N = nuclear fraction, *LE* = long exposure, *SE* = short exposure. Empty arrowheads indicate full-length proteins: red-rimmed = TBP 64Q, blue-rimmed = TBP 38Q, black-rimmed = endogenous human TBP (h_{eg}). Black arrowheads indicate the C-terminal TBP fragments (c1/ c2). **b.** Untransfected HEK 293T (HEK) cells were subjected to cytoplasmic-nuclear fractionation followed by western blotting. As representative members of the calpain system, calpain-1 (CAPN1), calpain-2 (CAPN2), and CAST were detected. The nuclear proteins lamin A and C, and cytoplasmic protein α-tubulin served as fraction purity controls. W = whole cell, C = cytoplasmic fraction, N = nuclear fraction. White circle (○) indicates an unspecific band. **c.** *In silico* nuclear export signal (NES) prediction in the human reference isoform 1 of TBP using

the NetNES tool. Graph shows the distribution of neural network (NN), hidden Markov model (HMM) and NES scores along the TBP sequence. Red line represents the cut-off value of 0.5. A schematic of TBP with its main features is depicted underneath the graph. Potential NES around amino acid Leu275 is indicated by a pink arrowhead. The predicted cleavage site cluster between Ala96 and Glu117 is marked with a red bracket

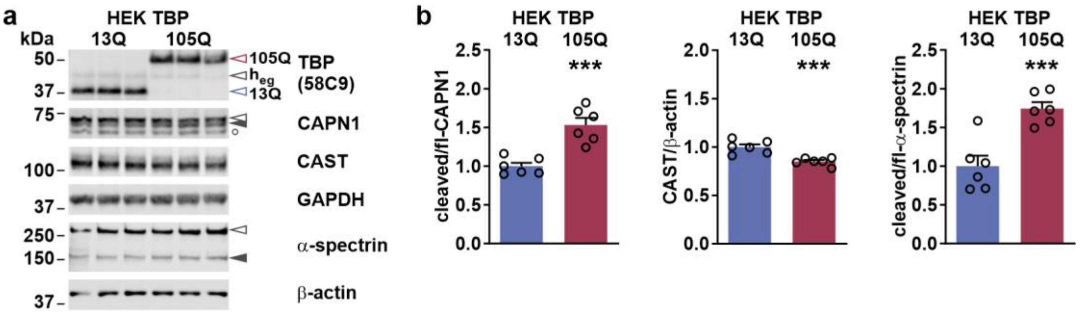

Suppl. Fig. S4 Calpain overactivation in HEK 293T cells expressing TBP 105Q. **a.** HEK 293T (HEK) cells expressing TBP with 13Q or 105Q were analyzed by western blotting. For assessment of calpain activation, samples were immunodetected with calpain-1 (CAPN1), CAST and α-spectrin antibodies. TBP was detected using antibody 58C9. Equal loading was confirmed using GAPDH and β-actin. Empty arrowheads indicate full-length proteins: black-rimmed = CAPN1, α-spectrin or endogenous human TBP (h_{eg}), red-rimmed = TBP 105Q, and blue-rimmed = TBP 13Q. Black arrowheads show respective breakdown products. White circle (○) indicates an unspecific band. **b.** Densitometric analysis shows CAPN1 cleavage ratio, CAST levels and α-spectrin cleavage ratio. Cleaved CAPN1 and α-spectrin levels were normalized to the respective full-length protein levels, and CAST levels to loading control β-actin. Obtained values were then normalized to the mean values of TBP 13Q-expressing HEK 293T cells. Bars represent means + SEM. n = 6 repeated transfections at different time points. ***$p \leq 0.001$ (Student's *t*-test)

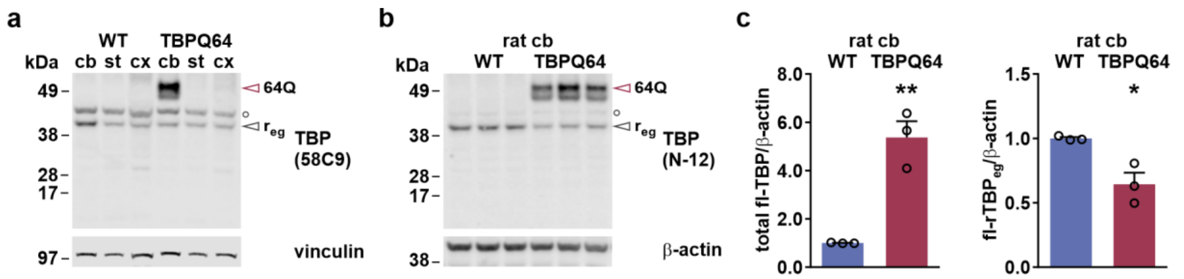

Suppl. Fig. S5 TBPQ64 striatum and cortex exhibits very low transgene expression and myc-TBP 64Q lowers endogenous TBP level. **a.** TBP expression was analyzed in cerebellum (cb), striatum (st) and cortex (cx) of TBPQ64 rats and WT controls by western blotting using the TBP 58C9 antibody. Equal loading was confirmed using vinculin. Empty arrowheads indicate full-length TBP: red-rimmed = 64Q, black-rimmed = endogenous rat TBP (r_{eg}). White circle (○) indicates an unspecific band. **b.** TBP expression analysis via western blotting was performed for cerebellum (cb) of TBPQ64 rats and WT controls using the TBP N-12 antibody. Equal loading was confirmed using β-actin. Empty arrowheads indicate full-length TBP: red-rimmed = 64Q, black-rimmed = endogenous rat TBP (r_{eg}). White circle (○) indicates an unspecific band. **c.** Densitometric analysis shows total levels of full-length (fl) TBP and levels of endogenous full-length rat TBP (fl-rTBP$_{eg}$) in cerebellum (cb). Total full-length or full-length rat TBP levels were normalized to loading control β-actin. Obtained values were then normalized to the mean values of WT cerebellum. Bars represent means + SEM. *n* = 3 animals per genotype. *$p \leq 0.05$, **$p \leq 0.01$ (Student's *t*-test)

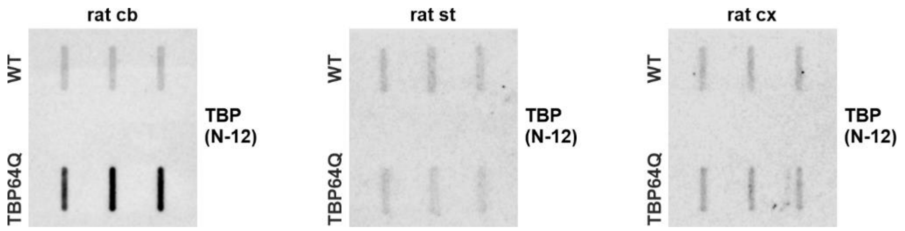

Suppl. Fig. S6 Only the cerebellum of TBPQ64 rats shows distinct occurrence of SDS-insoluble aggregates. Load of SDS-insoluble TBP aggregates in protein extracts of cerebellum (cb), striatum (st), and cortex (cx) of TBPQ64 rats and WT controls (*n* = 3 animals per genotype) was analyzed using filter retardation assays and the TBP N-12 antibody. Three animals were assayed per genotype

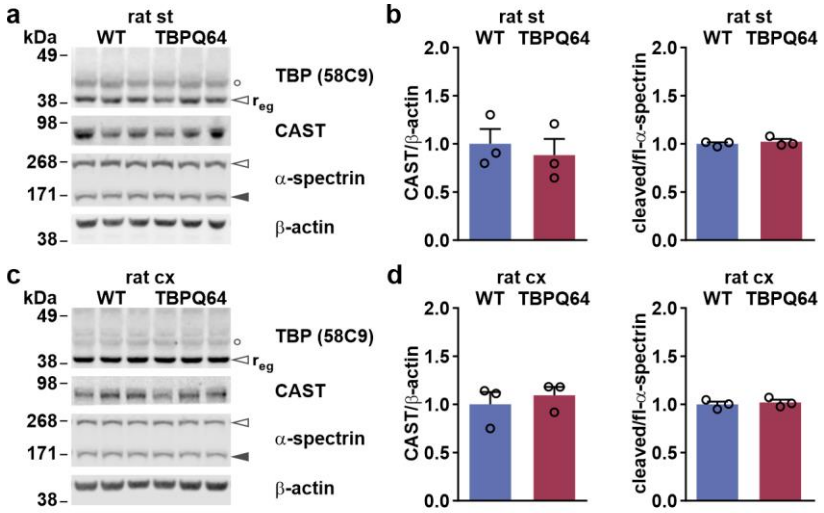

Suppl. Fig. S7 TBPQ64 striatum and cortex show no calpain overactivation. **a and c.** Calpain activation in striatum (st) and cortex (cx) of WT and TBPQ64 rats was investigated by western blotting and immunodetection using calpain-1 (CAPN1), CAST and α-spectrin antibodies. TBP was detected using antibody 58C9. Equal loading was confirmed using β-actin. Empty arrowheads indicate full-length endogenous rat TBP (r_{eg}) or α-spectrin. Black arrowheads show the calpain-dependent α-spectrin fragment. White circle (○) indicates an unspecific band. **b and d.** Densitometric analysis shows CAST levels and the α-spectrin cleavage ratio in striatum and cortex. CAST levels were normalized to loading control β-actin and cleaved α-spectrin levels to the respective full-length protein levels. Obtained values were then normalized to the mean values of WT striatum or cortex. Bars represent means + SEM. *n* = 3 animals per genotype

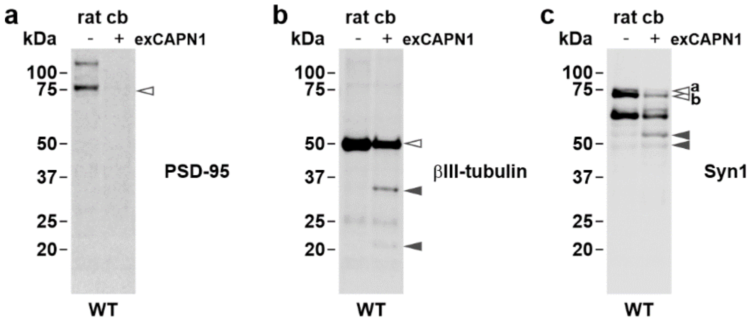

Suppl. Fig. S8 *In vitro* calpain cleavage assay of PSD-95 and βIII-tubulin. **a and b.** To test whether PSD-95 or βIII-tubulin represent calpain substrates, 25 µg protein extract of wild-type (WT) rat cerebellum was incubated with 200 ng of purified calpain-1 (exCAPN1) for 15 min. An undigested sample served as a negative control (-). After western blotting, PSD-95 and βIII-tubulin were detected. **c.** Cleavage of the known calpain substrate synapsin-1 (Syn1) was detected as an assay control. Black-rimmed arrowhead marks full-length proteins. Black arrowheads indicate protein fragments

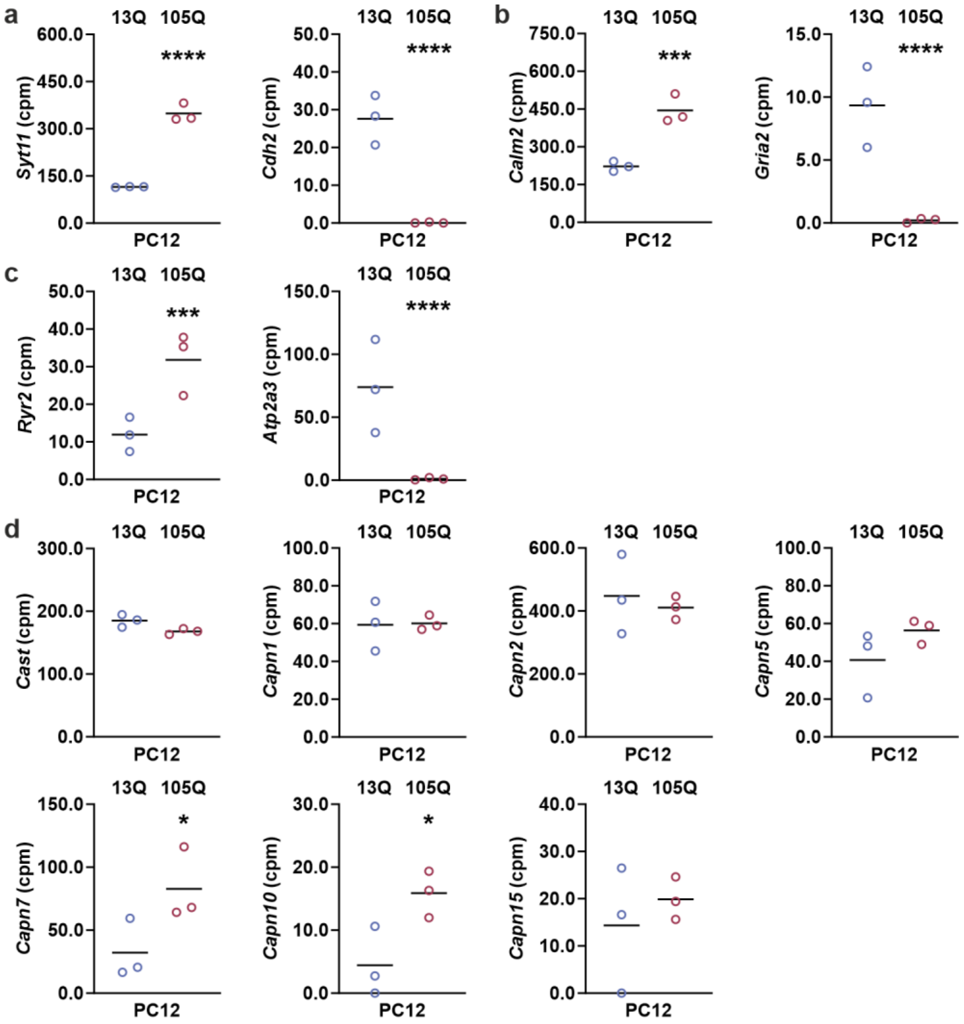

Suppl. Fig. S9 RNA sequencing of PC12 cells shows changes in representative genes of synaptogenesis and calcium pathways and but no major alterations in the expression of calpain genes. (**a-c**) Expression levels of the most dysregulated DEGs represented as counts per million (cpm) within the synaptogenesis (*Syt11*, *Cdh2*) (**a**) and calcium (*Ryr2*, *Atp2a3*) (**b**) pathways and shared by both pathways (*Calm2*, *Gria2*) (**c**). Horizontal lines represent means. n = 3 cell passages per genotype. ***p ≤ 0.001, ****p ≤ 0.0001 (adjusted p-values using the Benjamini-Hochberg procedure). (**d**) Expression levels of calpastatin (*Cast*) and members of the calpain family (*Capn1*, *-2*, *-5*, *-7*, *-10*, *-15*) as detected by 3' RNA sequencing of PC12 cells expressing TBP 13Q and 105Q are shown as counts per million (cpm) for each individual sample. Horizontal lines represent means. n = 3 cell passages per genotype. **p* ≤ 0.05 (adjusted *p*-value using the Benjamini-Hochberg procedure)

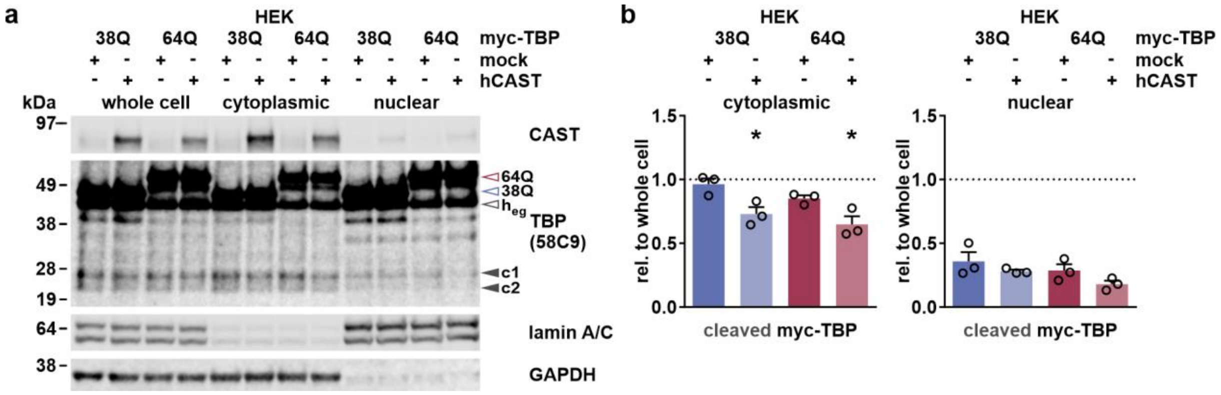

Suppl. Fig. S10 C-terminal TBP fragments c1 and c2 decrease in the cytoplasm upon CAST overexpression. **a.** HEK 293T (HEK) cells co-expressing myc-TBP 38Q or 64Q and human CAST for 72 h were subjected to cytoplasmic-nuclear fractionation, followed by western blotting analysis. Full-length TBP and its C-terminal fragments c1 and c2 were detected using antibody 58C9. Successful overexpression of CAST was confirmed using the respective antibody. The nuclear proteins lamin A and C, and cytoplasmic protein GAPDH served as fraction purity and loading controls. Empty arrowheads indicate full-length proteins: red-rimmed = TBP 64Q, blue-rimmed = TBP 38Q, black-rimmed = endogenous human TBP (h_{eg}). Black arrowheads indicate the C-terminal TBP fragments (c1/ c2). **b.** Densitometric analysis shows fragment c1/ c2 levels in the cytoplasmic and nuclear fraction upon overexpression of CAST. Levels were first normalized to fraction-specific loading control and then relative to (rel. to) whole cell as indicated by the dotted line. Bars represent means + SEM. $n = 3$ repeated transfections at different time points. *$p \leq 0.05$ (Student's *t*-test)

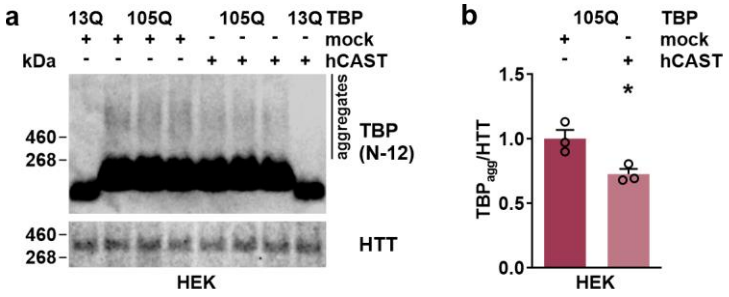

Suppl. Fig. S11 DD-AGE shows CAST overexpression-dependent lowering of polyQ-expanded TBP aggregation. **a.** Homogenates of HEK 293T (HEK) cells co-expressing TBP 13Q or 105Q and human CAST (hCAST) for 72 h were analyzed by DD-AGE. TBP and its high-molecular species (aggregates) were detected using antibody N-12. Huntingtin (HTT) served as loading control. **b.** Densitometric analysis shows reduction of TBP aggregates (TBP_{agg}) upon overexpression of CAST. TBP aggregate levels were first normalized to loading control HTT and then to the mean values of mock vector co-transfected HEK 293T cells expressing TBP 105Q. Bars represent means + SEM. $n = 3$ repeated transfections at different time points. *$p \leq 0.05$ (Student's *t*-test)
